# Healing of lytic lesions and restoration of bone health in multiple myeloma through sclerostin inhibition

**DOI:** 10.1186/s40164-025-00699-4

**Published:** 2025-08-22

**Authors:** Hayley M. Sabol, Aric Anloague, Japneet Kaur, Cecile Bustamante-Gomez, Sharmin Khan, Bethany C. Paxton, Mattie R. Nester, Jillian Hackney, Marta Diaz-delCastillo, Daniel Mann, Jeffrey B. Stambough, C. Lowry Barnes, Elena Ambrogini, Alison Frontier, Frank H. Ebetino, Syed Naqvi, Frits van Rhee, Christopher P. Wardell, Matthew T. Drake, Intawat Nookaew, Carolina Schinke, Maurizio Zangari, Jesus Delgado-Calle

**Affiliations:** 1 Department of Physiology and Cell Biology, University of Arkansas for Medical Sciences, 4301 W. Markham St., Little Rock, AR USA; 2 Winthrop P. Rockefeller Cancer Institute, University of Arkansas for Medical Sciences, Little Rock, AR USA; 3 Division of Endocrinology and Metabolism, University of Arkansas for Medical Sciences, Little Rock, AR USA; 4 Forensic Medicine, University of Aarhus, Aarhus, Denmark; 5 Department of Biomedical Informatics, University of Arkansas for Medical Sciences, Little Rock, AR USA; 6 Department of Orthopedic Surgery, University of Arkansas for Medical Sciences, Little Rock, AR USA; 7 Center for Musculoskeletal Disease Research, University of Arkansas for Medical Sciences, Little Rock, AR USA; 8 Central Arkansas Veterans Healthcare System, Little Rock, AR USA; 9 Department of Chemistry, University of Rochester, Rochester, NY USA; 10 Biovinc LLC, Pasadena, CA USA; 11 Myeloma Center, University of Arkansas for Medical Sciences, Little Rock, AR USA; 12 Department of Internal Medicine, Mayo Clinic, Rochester, MN USA

**Keywords:** Multiple myeloma, Wnt, Sclerostin, Bone, Lytic, Repair, Tumor

## Abstract

**Background:**

Multiple myeloma (MM) is associated with a debilitating bone disease that poses significant therapeutic challenges. MM bone disease is characterized by increased bone resorption and suppression of osteoblasts, which hinders the repair of damaged bone. Sclerostin, an antagonist of Wnt signaling, is elevated in MM patients, and its inhibition with a neutralizing antibody (Scl-ab) has been shown to restore osteoblast function in mouse models of MM. However, it remains unclear whether Scl-ab can promote skeletal repair, enable effective tumor control when combined with anti-cancer agents, or improve bone health in MM patients.

**Methods:**

To investigate these knowledge gaps, we used preclinical MM mouse models and patient-derived samples. We also characterize the impact of Scl-ab on cancer and osteoblastic cells isolated from mouse models through bulk and single-cell RNA sequencing. Lastly, we performed a retrospective analysis of the efficacy of Scl-ab to improve bone health in patients with MM in remission.

**Results:**

Scl-ab promoted skeletal repair and enabled tumor suppression by an anti-cancer agent in various animal models of established MM bone disease. MM tumors suppressed Wnt signaling and decreased the number of osteoblasts and osteo-CAR cells, and treatment with Scl-ab reversed these effects. Treatment with Scl-ab increased bone mass and repaired bone in patients with MM in remission, even when combined with maintenance chemotherapy.

**Conclusions:**

Our findings highlight the potent bone-healing effects of Scl-ab and its potential as an adjuvant to anti-cancer therapy, offering a promising approach to improve clinical outcomes and the quality of life for MM patients.

**Supplementary Information:**

The online version contains supplementary material available at 10.1186/s40164-025-00699-4.

## Background

Multiple myeloma (MM) is the second most common hematologic cancer in the United States [1], characterized by the clonal proliferation of malignant plasma cells within the bone marrow [2]. Bone disease is a hallmark of MM, affecting 80% of newly diagnosed patients [3] and contributes significantly to morbidity and mortality [4, 5]. Current therapies primarily target osteoclasts and reduce the risk of skeletal complications, but they do not repair damaged bone, which continues to deteriorate as the disease progresses [6, 7]. Therefore, effective management of bone health is crucial to improving clinical outcomes in MM patients.

MM-induced bone disease is shaped by interactions between tumor cells and cells of the bone marrow microenvironment, which support bone destruction by increasing osteoclasts and lead to the formation of debilitating lytic lesions [8]. In addition, MM cells and cells of the tumor microenvironment secrete Wnt antagonists, such as Dkk1 [9] and sclerostin [10, 11], which suppress osteoblast function and hinder bone repair, highlighting the need to restore osteoblast function to repair damaged bone in MM patients. Prior work has shown that blocking sclerostin with neutralizing antibodies (Scl-ab) restores osteoblast function and increases bone mass in MM mouse models [10, 12, 13], but the tumor growth persists unchecked. However, several critical questions remain regarding Scl-ab treatment in MM. These include whether it can heal lytic lesions and restore bone microarchitecture, the cellular and molecular mechanisms by which it promotes bone formation in the inhibitory MM niche, and its efficacy when used in conjunction with anti-tumor therapy. Moreover, although sclerostin levels are increased in MM patients, the effects of Scl-ab on bone health in these individuals remain unexplored.

In this study, we show in preclinical MM models that romosozumab [14], a clinically approved Scl-ab, repairs damaged bone, activates Wnt signaling and targets specific subpopulations of osteoblastic cells, and its bone anabolic efficacy is not affected when combined with anti-tumor therapy. Furthermore, we show for the first time that Scl-ab increases bone mass and repairs damaged bone in patients with MM in remission. The results from this study warrant further investigation to assess the potential of romosozumab as an adjunctive treatment to improve bone health in MM patients.

## Methods

Here, we provide a brief description of the methods used; for more detailed information, please refer to the Supplementary Methods.

**Reagents.** The bone-targeted GSI (BT-GSI) [15] is a conjugate formed by (i) a modified, less active bisphosphonate moiety with high bone affinity designed to direct the conjugate to the skeleton; (ii) a pH-sensitive labile linker that binds the cargo; and (iii) the cargo, the small molecule γ-secretase inhibitor (GSI) XII that inhibits Notch signaling by preventing the cleavage of the Notch intracellular domain of Notch receptors. Detailed chemical steps for generating BT-GSI were described before [15].

**Animal studies.** 7-week-old immunocompetent C57BL/KaLwRijHsd (RRID: MGI:2164290) mice were injected intratibially with 1 × 10^5^ 5TGM1 MM cells or saline. After 4 weeks, mice were randomized by tumor burden and cancellous bone mass to groups (i) vehicle (saline), (ii) 100 mg/kg; 1x/week Scl-ab (i.p.), (iii) 2.5 mg/kg; 3x/week BT-GSI (i.p.), or (iv) Scl-ab + BT-GSI (combo) combined for 4 additional weeks. These dosing regimens were selected based on prior studies [12, 15, 16]. 7-week-old immunodeficient NSG (RRID: IMSR_JAX:005557) mice were injected intravenously with 5 × 10^5^ OPM2-Luc^+^ MM cells or saline. After one week, mice were randomized based on tumor burden and cancellous bone mass to groups (i) saline, (ii) Scl-ab, (iii) BT-GSI (i.p.), or (iv) combo combined for 3 additional weeks. To assess survival, the health of the mice was monitored daily, and mice were euthanized at the first signs of back leg paralysis. Toxicological studies are described in the Supplementary Materials.

Ex vivo **organ cultures.** Ex vivo MM-human bone organ cultures were established using human cancellous bone fragments similar in size obtained from femoral heads discarded after hip arthroplasty, as described before [17, 18]. Bone fragments and 2 × 10^5^ OPM2 MM cells were incubated for 24 h. After 24 h, bones with infiltrated MM cells were transferred to a new plate with fresh media and treated with vehicle, BT-GSI (10 μm), Scl-ab (15 μm), or a combination of BT-GSI + Scl-ab. Treatments were refreshed every 3 days, and conditioned media was collected after 4 or 11 days. Flow cytometry studies are described in the Supplementary Materials.

**scRNA Sequencing.** 7-week-old NSG mice were injected with 5 × 10^5^ OPM2 mCherry^+^ MM cells, saline, or treated with Scl-ab (100 mg/kg; 1x/week). After 2 weeks of treatment, a time point at which MM-induced bone loss and Scl-ab-induced bone anabolic effects are both evident, the muscle, periosteum, and epiphyses were removed from the tibias and femurs. The final dose of Scl-ab was administered 10 h before tissue collection. The bones were cut longitudinally and processed to obtain osteoblastic cell populations, as reported before [19]. Cells from 2 to 4 mice per group were pooled, stained with DAPI (1ug/mL), and sorted using FACS to remove mCherry^+^ MM cells or dead cells from the sample. Single-cell analysis was performed following our bioinformatic pipelines [19, 20].

**Bulk RNA sequencing.** 8-week-old immunocompetent C57BL/KaLwRijHsd mice were injected intravenously with 1 × 10^6^ GFP^+^ 5TGM1 MM cells. After 2 weeks, mice were treated with vehicle, BT-GSI, Scl-ab, or BT-GSI + Scl-ab. Mice were sacrificed after 3 weeks. The bone marrow from 3 long bones was flushed out, and RNA was obtained from MM cells isolated using CD138^+^ microbeads (Miltenyi Biotec, cat #. 130-098-257). Preparation of RNA sequencing libraries (mRNA) and transcriptome sequencing was conducted by Novogene Co., LTD (San Jose, CA, USA).

**Human samples and histological analysis.** Bone biopsies of 3-mm diameter from patients with MGUS or newly diagnosed MM (*n* = 12–14) were retrieved from Danish histopathological biobanks and processed for RNA in situ hybridization as described before [18].

**Patient population.** This retrospective study involved patients with MM (*n* = 5, 4/1 F/M, 71.8 years old (67–86)) in complete remission and with osteoporosis and a high risk of fracture, who received romosozumab (210 mg monthly) therapy at the University of Arkansas for Medical Sciences (UAMS) (*n* = 3) or the Mayo Clinic (*n* = 2) for 12 months. Patients treated at UAMS had not received anti-resorptive therapy prior to romosozumab, whereas patients treated at the Mayo Clinic had received bisphosphonates, with the last dose administered 6 to 14 months before starting romosozumab. Contemporaneous UAMS MM patients in remission (*n* = 10, 10/0 F/M, average 74 years old (64–83) who did not receive romosozumab and had available DXA (*n* = 3) or PET-CT scans (*n* = 10) with similar time intervals between scans served as control subjects. Nine of the ten control patients received bisphosphonates during the data collection. Medical records were accessed to gather data encompassing demographic information, baseline disease characteristics, therapies, PET-CT and DXA scans, and laboratory parameters. Disease remission was determined according to the International Myeloma Working Group (IMWG) criteria. Table S2 summarizes the patients’ characteristics. Femoral neck BMD measurements were made by DXA (Lunar Prodigy System; GE Healthcare). DXA scans were evaluated according to the International Society of Clinical Densitometry criteria (www.iscd.org/visitors/positions/OPReferences.cfm). PET-CT scans from multiple myeloma patients were retrieved and processed as described in the Supplementary Materials.

**Statistical analysis.** Data were analyzed using GraphPad Prism (GraphPad Software, Inc., San Diego, CA, USA). Differences in means were analyzed using a combination of unpaired and paired *t*-test, One-Way ANOVA, and Two-Way ANOVA RM tests, followed by pairwise multiple comparisons (Tukey post hoc test). Values were reported as means ± SD, unless otherwise indicated in the figure legends.

## Results

### Anti-sclerostin preserves the efficacy of anti-tumor therapy

**Fig. 1:**
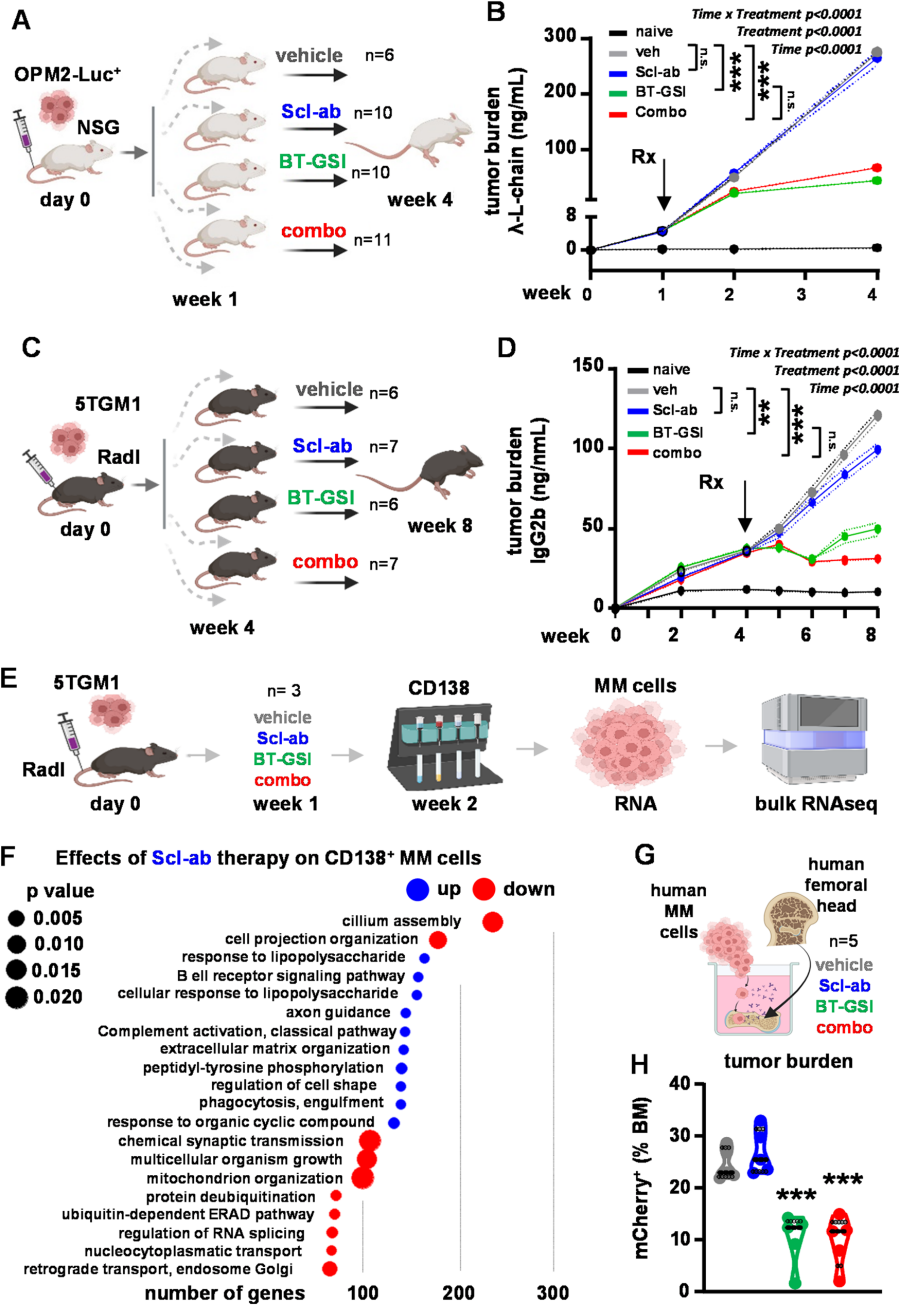
Scl-ab enables suppression of tumor growth when co-administered with anti-cancer therapy in mice with established multiple myeloma. (**A**) Human xenograft mouse model experimental design. (**B**) Serum human lambda light chain paraprotein levels (mean ± SE). *n* = 6–11 mice/group. (**C**) Immunocompetent mouse model experimental design. (**D**) Serum murine IgG2b paraprotein levels (mean ± SE). *n* = 6–7 mice/group. Experimental design (**E**) and gene ontology (GO) enrichment analysis (**F**) in 5TGM1 MM cells isolated from mice receiving vehicle vs. Scl-ab. *n* = 3 mice/group. (**G**) Ex vivo bone-MM organ cultures were established with bone fragments from a control subject and human OPM2 MM cells. (**H**) Percentage of mCherry^+^ MM cells in human bone organ cultures treated with vehicle, BT-GSI, Scl-ab, or combo. *n* = 5/group. *p < 0.05; **p < 0.01; ***p < 0.001 vs. MM-vehicle by Two-Way ANOVA RM (B - week 4, D - week 8) or by One-Way ANOVA (H). Rx = treatment initiation; n.s.=non-significant

Since Scl-ab does not affect tumor growth [10, 12, 13], we first evaluated the impact of co-administering Scl-ab on the efficacy of anti-cancer therapy in tumor progression. Human OPM2-Luciferase^+^ MM cells, which harbor the t(4;14) translocation linked to poor prognosis, were injected into the tail vein of immunodeficient mice. After 1 week, mice were treated with a bone-targeted Notch inhibitor (BT-GSI), which has potent anti-MM effects [15], or Scl-ab either as single agents or in combination (combo) for 3 weeks (Fig. 1A). Mice receiving vehicle or Scl-ab showed a similar exponential increase in tumor growth, as indicated by elevated serum paraprotein levels (Figs. 1B). In contrast, treatment with BT-GSI or the combo resulted in a similar 80% reduction in tumor burden compared to the vehicle-treated group and prolonged survival (Fig. S1A-B). Similar tumor reductions were seen with quantitative assessment of bioluminescence in bone (Fig. S1C-F). Of note, no changes in spleen weight were observed with any of the therapies (Fig. S1G). Next, we injected 5TGM1 murine MM cells directly into the tibia of immunocompetent mice. After four weeks, treatment with BT-GSI, Scl-ab, or combo was initiated for an additional four weeks (Fig. 1C). While Scl-ab alone did not impact tumor progression, both BT-GSI and combo significantly reduced tumor growth by 70% relative to the vehicle-treated group (Fig. 1D), without significant changes in spleen weight (Fig. S1H-I). Scl-ab induced a modest increase in glucose and a decrease in hemoglobin levels, but no other significant toxicities were observed when administered alone or in combination with BT-GSI (Table S1).

To gain mechanistic insights into the effects of these treatments on tumor cells, we isolated CD138^+^ 5TGM1 MM cells from the bone marrow of mice treated with veh, BT-GSI, Scl-ab, or combo for one week and performed bulk RNA-seq analysis (Fig. 1E; Fig. S2A-C). Treatment with BT-GSI (Fig. S3A; Data file S1) or the combo (Fig. S3B; Data file S2) led to the upregulation of apoptosis pathways and downregulation of cell cycle, DNA repair, and cell division biological processes, and downregulated the expression of Notch pathway targets and genes associated with cell proliferation in CD138^+^ MM cells compared to MM cells from the vehicle group (Figs. S3C-D). No significant changes were observed in apoptotic or cell cycle pathways or in the expression of Notch- or proliferation-related genes with Scl-ab (Fig. 1F and Data file S3). To further investigate the anti-tumor efficacy of the Scl-ab in combination with anti-cancer therapy within a human bone microenvironment, we established ex vivo organ cultures using human bone specimens infiltrated with human OPM2 MM cells, a system that recapitulates the tumor microenvironment in patients [21] (Fig. 1G). In line with the findings in mouse models, Scl-ab did not affect the growth of MM cells. In contrast, BT-GSI alone or in combination with Scl-ab reduced the proportion of MM cells in bone by 50% compared to the vehicle group (Fig. 1H).

### Anti-sclerostin treatment repairs damaged bone in MM

**Fig. 2:**
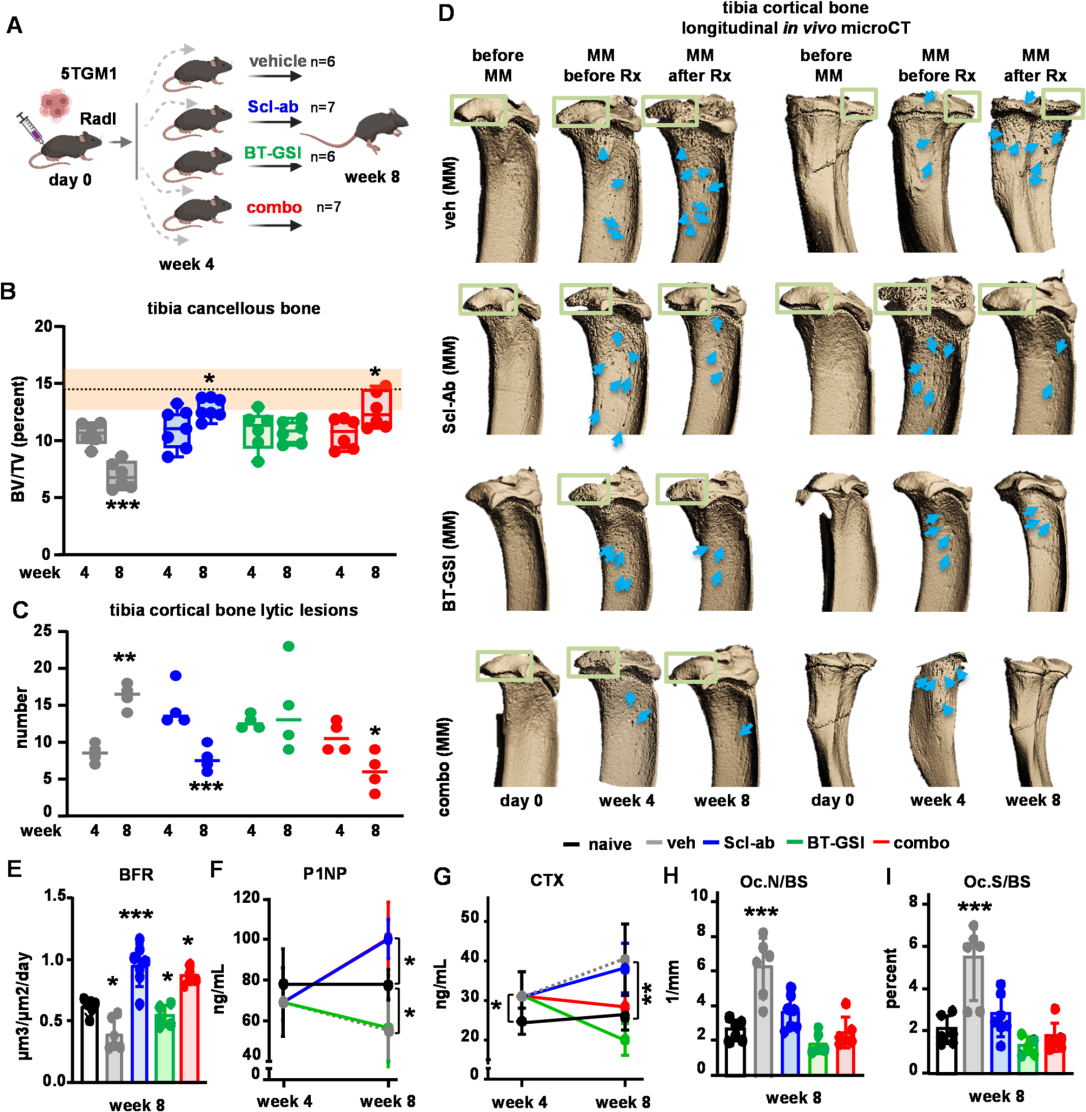
Scl-ab repairs damaged bone in immunocompetent mice with established MM-induced bone disease. (**A**) Immunocompetent mouse model experimental design. Quantification of tibiae (**B**) cancellous BV/TV, (**C**) cortical lytic lesions, and (**D**) representative microCT 3D reconstruction images before (week 4) and after (week 8) treatment, (**E**) bone formation rate in cancellous bone, (**F**) serum P1NP levels, (**G**) serum CTX levels, (**H-I**) osteoclasts number and surface in cancellous bone from immunocompetent mice injected intratibially with 5TGM1 MM cells and treated with veh, Scl-ab, BT-GSI or combo for 4 weeks. *n* = 6–7 mice/group. BFR/BS = bone formation rate per bone surface; Oc.N/BS = osteoclast number per bone surface; Oc.S/BS = osteoclast surface per bone surface. *p < 0.05; **p < 0.01; ***p < 0.001 vs. vehicle by paired t-Test (**B**-**C**) or One-Way ANOVA (**E**-**I**). The dotted line and orange color indicate basal (before MM injection) BV/TV mean ± SD values. Blue arrows and green squares indicate lytic lesions (**D**)

Next, we investigated the effects of Scl-ab on MM-induced bone disease in bones using the mouse models described above. We hypothesized that combining Scl-ab with anti-tumor therapy could, in addition to inhibiting tumor growth, concurrently reduce bone destruction and promote bone formation. To assess whether the Scl-ab therapy could repair damaged bone induced by MM tumors, we performed longitudinal in vivo micro CT imaging at multiple time points: baseline (before injections), before treatment (week 4), and after 4 weeks of treatment (week 8) in a murine model where 5TGM1 MM cells were injected intratibially (Fig. 2A), an approach that allows tracking changes in bone mass and lytic lesions within each mouse over time. Mice receiving vehicle showed progressive bone loss (20% at week 4 and 50% at week 8; Fig. 2B). Scl-ab restored bone mass to baseline levels and repaired lytic lesions (Figs. 2B-D and Figs. S4A-C), improved key cancellous bone formation indexes to those seen in naïve mice, and decreases cancellous osteoclast numbers/surface to control levels (Figs. 2E-I and Figs. S4D-F). BT-GSI administered alone prevented further bone loss by inhibiting resorption, an effect due to the inhibitory actions of GSI on RANKL expression and osteoclast differentiation [15], but did not improve bone mass or repair existing lytic lesions. The beneficial skeletal effects of Scl-ab were preserved when combined with anti-cancer therapy BT-GSI.

**Fig. 3:**
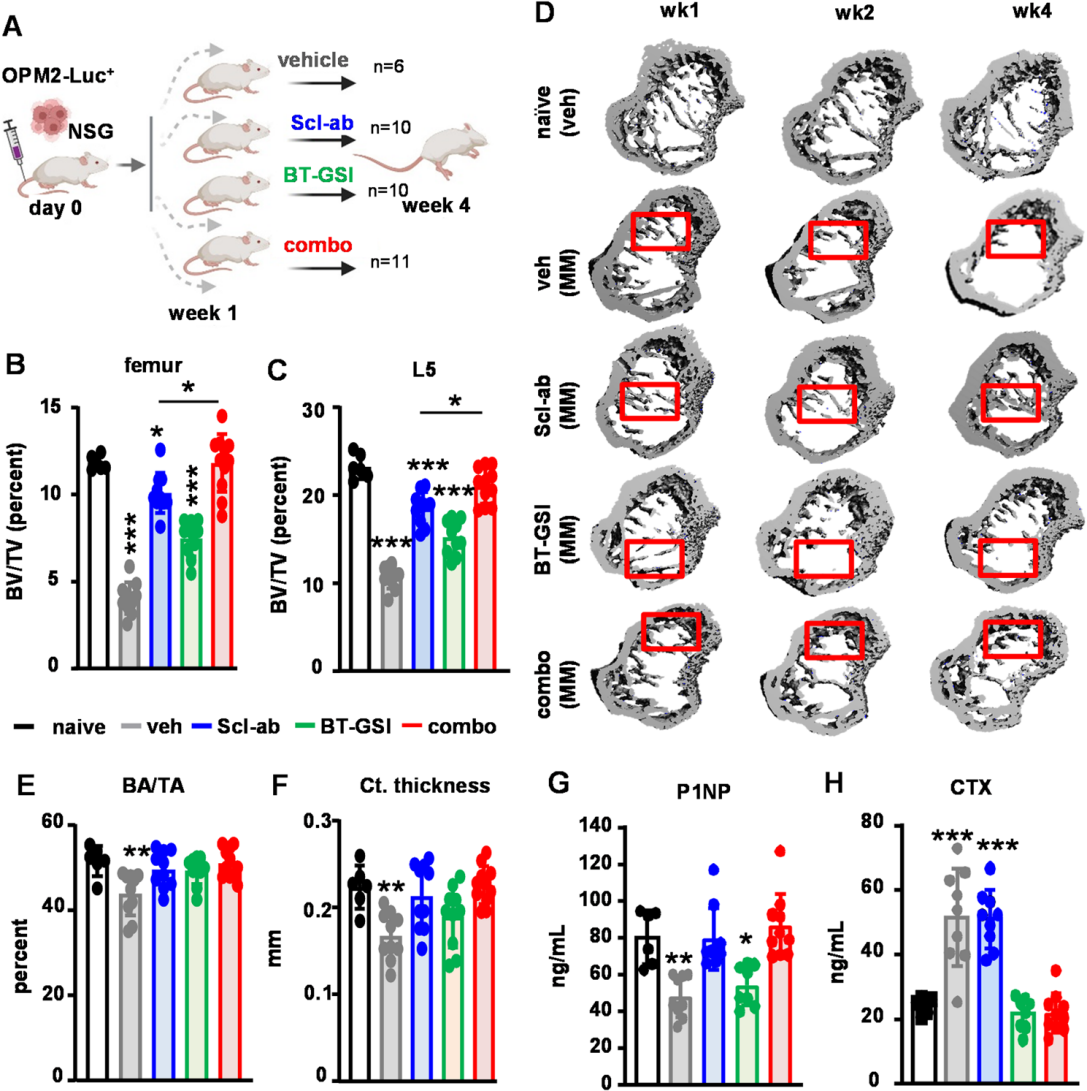
Scl-ab improves bone health in a xenograft mouse model of established MM-induced bone disease. (**A**) Human xenograft mouse model experimental design. (**B-C**) Femur and L5 vertebrae bone volume/tissue volume (BV/TV, week 4), (**D**) representative microCT 3D reconstruction longitudinal images of tibiae cancellous BV/TV, (**E-F**) femur cortical (Ct) bone area/tissue area (BA/TA) and thickness, and (**G-H**) serum markers of bone formation P1NP and bone resorption CTX (week 4) from immunodeficient mice injected with human OPM2 MM cells in the tail vein and treated with vehicle, Scl-ab, BT-GSI, or combo for 3 weeks. *n* = 6–11 mice/group. *p < 0.05; **p < 0.01; ***p < 0.001 vs. vehicle by One-Way ANOVA. Red squares indicate areas of bone repair (**D**)

MicroCT analysis revealed significant improvements in cancellous bone mass, trabecular number, thickness, and separation in the femurs and vertebrae and femoral cortical bone volume and thickness (Figs. 3A-F and Figs. S5), and restoration of cancellous bone mass in the tibiae (Fig. 3D) of immunodeficient mice bearing OPM2 MM cells treated with Scl-ab as a single agent. In this experiment, the combination treatment group exhibited higher cancellous bone mass compared to the group treated only with Scl-ab. While Scl-ab alone increased serum levels of P1NP and BT-GSI alone decreased the bone resorption marker CTX, the combination therapy resulted in a simultaneous increase in serum P1NP and a reduction in CTX (Figs. 3G-H).

**Fig. 4:**
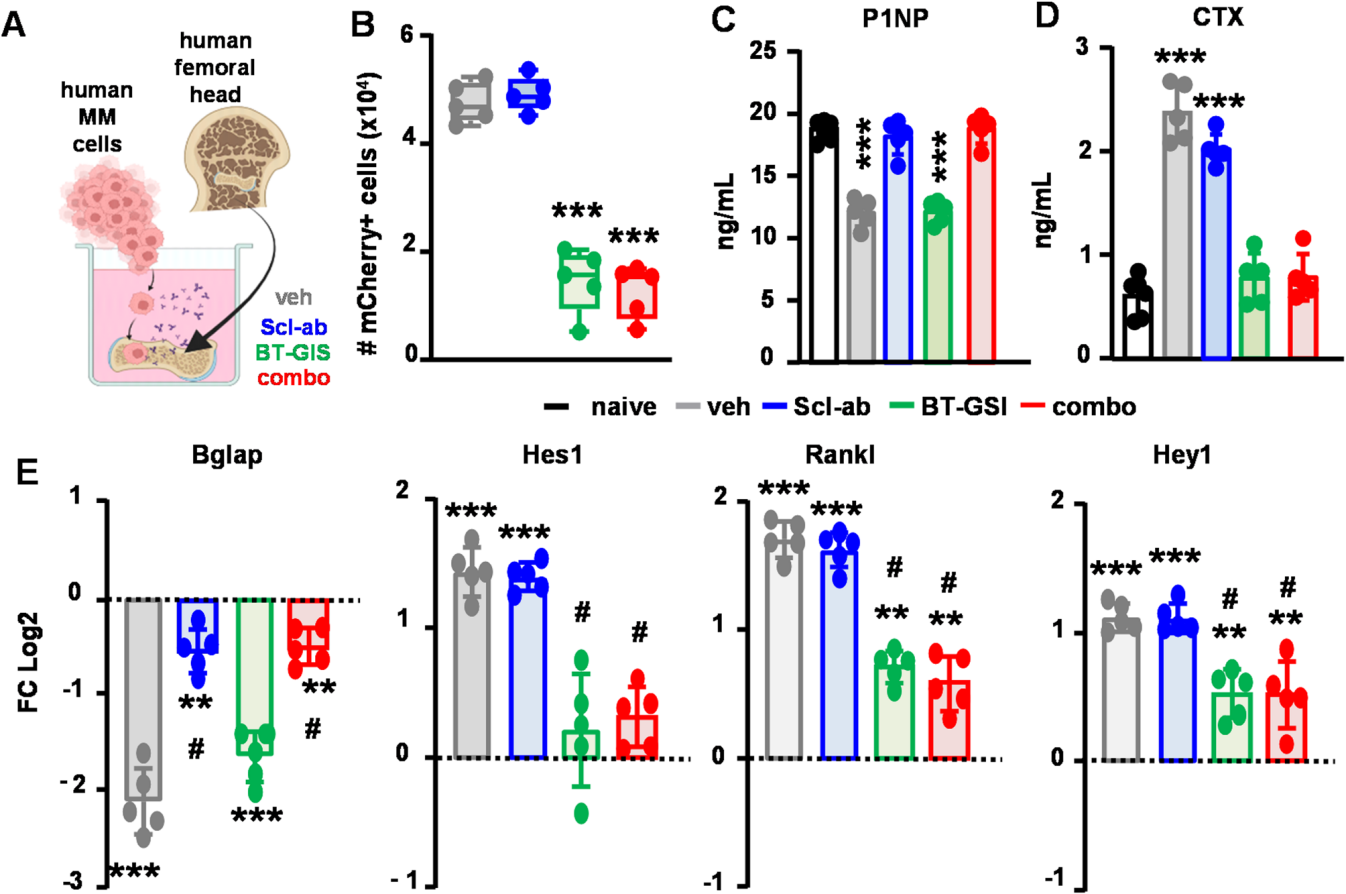
Impact of Scl-ab on human bones bearing human MM tumors cultured ex vivo. **(A)** Ex vivo bone-MM organ cultures were established using bone fragments from a control subject and human OPM2 MM cells and treated with vehicle, Scl-ab, or combo. Number of Cherry + OPM2 MM cells (**B**), levels of P1NP **(C)** and CTX **(D)** in conditioned media, and **(E)** gene expression of *BGLAP*, *HES1*, *RANKL*, and *HEY1*. *n* = 5 bones/group. *p < 0.05; **p < 0.01; ***p < 0.001vs. saline and #*p* < 0.05 vs. MM vehicle by One-way ANOVA

We further evaluated the effects of Scl-ab on human bone in ex vivo organ cultures (Fig. 4A). Consistent with our in vivo observations, treatment with BT-GSI, with or without Scl-ab, significantly reduced the number of MM cells growing in bone organ cultures, while Scl-ab alone did not affect MM cell number (Fig. 4B). After 14 days, MM cells significantly reduced P1NP and increased CTX levels in the culture media, downregulated the expression of the osteoblast marker *BGLAP*, and upregulated the expression of the pro-osteoclastogenic cytokine *RANKL* and the Notch target genes *HES1* and *HEY1* (Fig. 4C-E). Scl-ab treatment increased P1NP levels and *BGLAP* expression but did not affect CTX or *RANKL/Notch* expression, which remained elevated compared to the control group. BT-GSI decreased CTX, *RANKL*, and Notch target genes compared to controls but did not affect P1NP or *BGLAP*, which remained decreased compared to controls. Remarkably, the combination therapy simultaneously increased P1NP and *BGLAP* levels while decreasing CTX, *RANKL*, and Notch gene expression (Figs. 4C-E). Similar results were observed in bone tissue from a different donor (Fig. S6). Together, these in vivo and ex vivo findings demonstrate that Scl-ab repairs damaged bone and improves bone health by enhancing bone formation, and that this bone protective effect is preserved when co-administered with anti-cancer therapy.

### Scl-ab reverses the suppression of osteoblasts and osteo-CAR cells by MM tumors

**Fig. 5:**
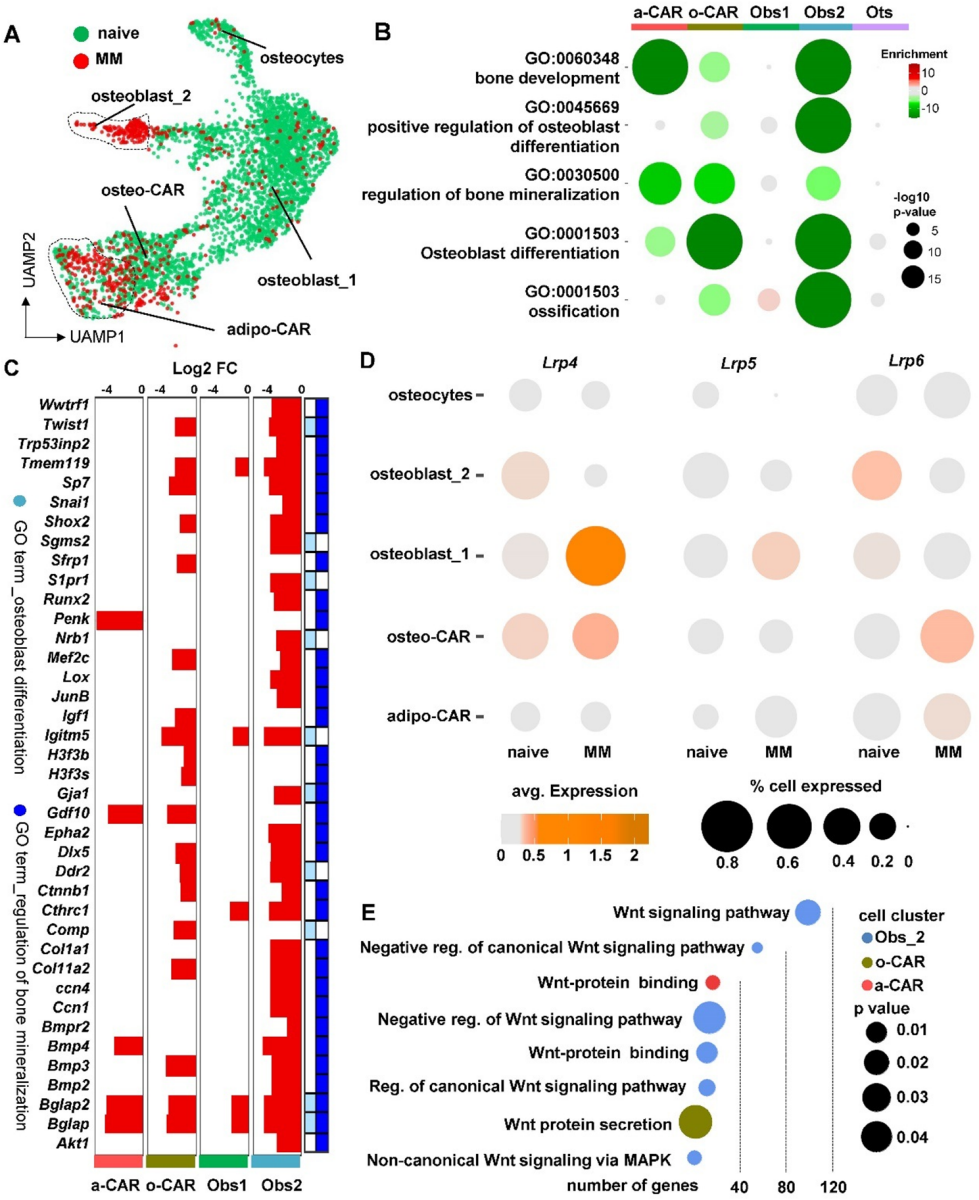
Single-cell transcriptomic profiling identifies osteoblasts and osteo-CAR cells as targets of MM tumors. (**A**) Uniform Manifold Approximation and Projection (UMAP) plot representations of osteoblastic cells isolated from control (naïve) or mice injected with human OPM2 MM cells in the tail vein showing five clusters: osteocytes (Ots), osteoblasts_1 (Obs_1), and osteoblasts_2 (Obs_2), osteo-CAR, and adipo-CAR cells. (**B**) Gene ontology (GO) enrichment analysis in downregulated genes expressed in osteoblastic cells from naïve vs. MM mice. Bubble size is proportional to the enrichment p-value, and color intensity is proportional to enrichment. (**C**) Gene expression plot of downregulated genes in GO terms 001503 and 0030500 according to their relative abundance (log2 fold change) and statistical value (-log10 p-value) for each cell cluster in naïve vs. MM mice. (**D**) Expression of *Lrp4*, *Lrp5*, and *Lrp6* in osteoblastic cells from naïve vs. MM mice. Bubble size is proportional to the percentage of cells expressing the gene, and color intensity is proportional to the expression level of each cell type. (**E**) Downregulated pathways associated with Wnt signaling in osteoblastic cells from naïve vs. MM mice. Bubble size is proportional to the enrichment p-value, and color intensity is proportional to the cell cluster

Next, we utilized single-cell RNA sequencing to further characterize the impact of MM and Scl-ab on osteoblastic cells. Osteoblastic cells were isolated from the bones of naïve mice, mice injected with human OPM2 MM cells, and naïve mice treated with Scl-ab. We identified five distinct cellular clusters based on gene expression patterns: [1] osteocytes [2], osteoblasts_1 [3], osteoblasts_2 [4], osteo-Cxcl12-abundant-reticular (CAR), and [5] adipose-CARs (Figs. S7A-C). MM cells led to a reduction of osteocyte, osteoblast_1, and osteo-CAR populations compared to naïve mice, while no significant changes were observed in the adipo-CAR population (Fig. 5A and S7D). Notably, our analysis also detected a unique subpopulation of “diseased” osteoblasts (osteoblasts_2) that remained in the presence of tumors.

Gene ontology analysis (see Data files S4-8 for top up/down GO terms and genes) of terms associated with bone biology revealed downregulation of pathways related to bone development and bone mineralization, with the most pronounced effects observed in osteoblasts_2 and osteo-CAR compared to adipo-CAR cells (Fig. 5B). No major changes in these pathways were observed in osteocytes or osteoblast_1 populations, likely due to the low number of cells retrieved in the MM group. Both osteoblasts_2 and osteo-CARs exhibited consistent downregulation of key genes related to osteoblast differentiation and regulation of bone mineralization, while adipo-CARs or osteoblasts_1 were less affected by MM (Fig. 5C). Furthermore, both populations of osteoblasts and osteo-CAR cells expressed higher levels of the sclerostin co-receptor *Lrp4* in naïve mice (Fig. 5D), which were upregulated by MM tumors in osteoblasts_1 and osteo-CARs, and decreased in osteoblast_2. Moreover, we noted the downregulation of Wnt-related gene ontology terms in osteoblasts_2 and, to a lesser extent, in osteo-CARs in the MM group (Fig. 5E).

**Fig. 6:**
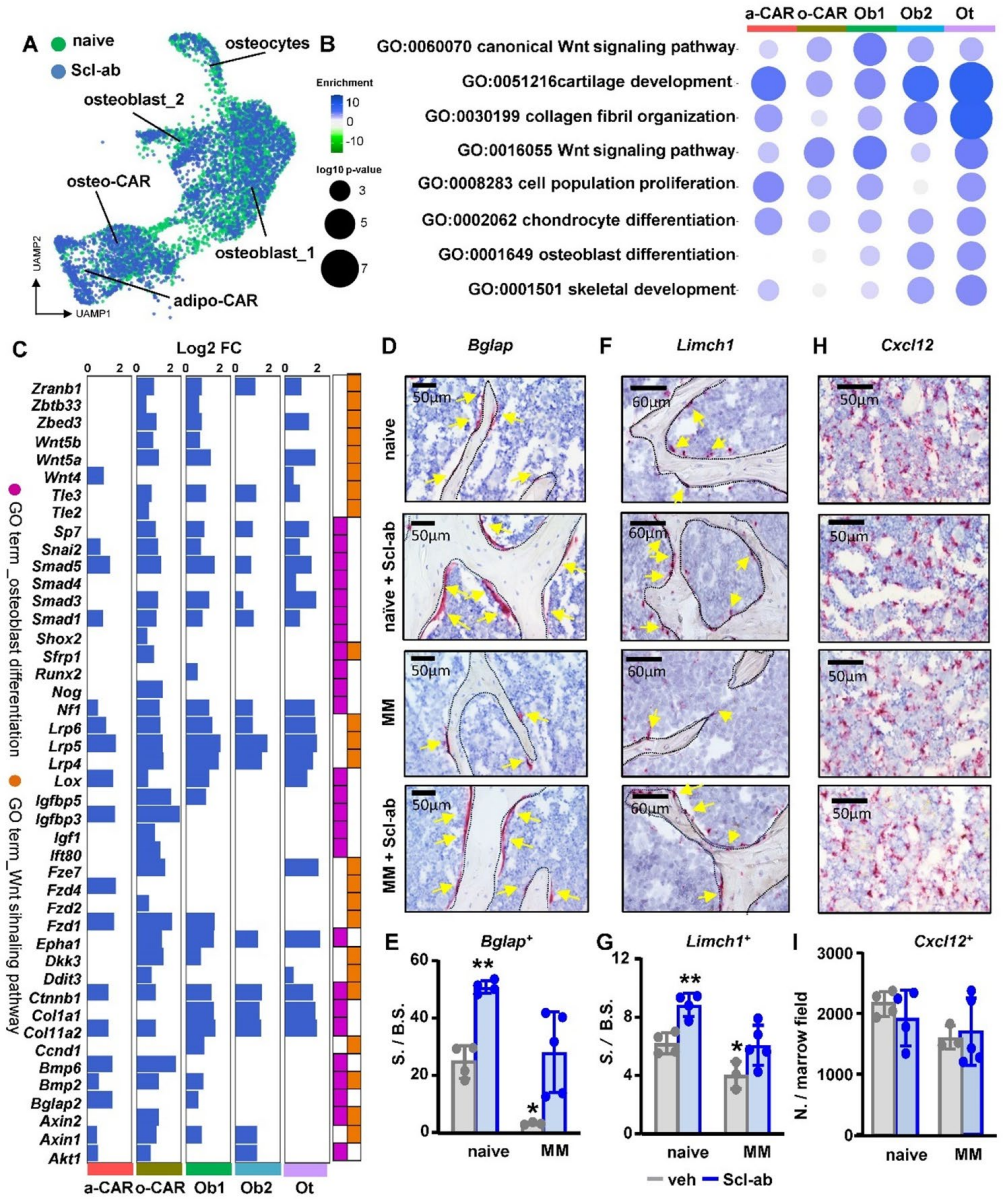
Single-cell transcriptomic profiling shows that Scl-ab increases osteoblasts and osteo-CAR cells in the bone. (**A**) Uniform Manifold Approximation and Projection (UMAP) plot representations of osteoblastic cells isolated from naïve and naïve mice treated with anti-sclerostin-antibody (Scl-ab) showing 5 clusters: osteocytes (Ots), osteoblasts_1 (Obs_1), and osteoblasts_2 (Obs_2), osteo-CAR, and adipo-CAR cells. (**B**) Gene ontology (GO) enrichment analysis in upregulated genes expressed in osteoblastic cells from naïve vs. Scl-ab-treated mice. Bubble size is proportional to the enrichment p-value, and color intensity is proportional to enrichment. (**C**) Gene expression plot of upregulated genes in GO terms 0001649 and 0060070 according to their relative abundance (log2 fold change) and statistical value (-log10 p-value) for each cell cluster in naïve vs. Scl-ab treated mice. RNAscope representative images (**D**,** F**,** H**) and quantification (**E**,** G**,** I**) of *Bglap*^+^ cells on the bone surface, *Limch1*^+^ cells on the bone surface, and *Cxcl12*^+^ cells in the marrow from L4-6 vertebrae from control (naïve), naïve mice treated with Scl-ab, mice bearing human OPM2 MM cells receiving vehicle, or mice bearing murine OPM2 MM cells receiving Scl-ab. *n* = 3–5 bones/group *p < 0.05; **p < 0.01 vs. naive by One-Way ANOVA

Analysis of the Scl-ab scRNAseq dataset (Fig. 6A; see Data files S9-13 for top up/down GO terms and genes) revealed upregulation of GO terms associated with Wnt signaling, cell proliferation, osteoblasts and chondrocyte differentiation, collagen formation, and skeletal development across the different cell clusters from Scl-ab treated mice compared to naïve mice (Fig. 6B). This upregulation was also seen in genes involved in osteoblast differentiation and the Wnt signaling pathway, particularly in osteo-CAR cells and osteoblasts_2, with more modest changes observed in adipo-CARs (Fig. 6C). To further study the effects of MM and Scl-ab on osteoblastic cells, we quantified osteoblasts (*Bglap*^+^) and osteo-CARs (*Limch*1^+^), which are primarily located at bone surfaces [19], adipo-CARs (*Cxcl1*2+), which are mainly found within the bone marrow near sinusoidal blood vessels [22], and MM cells (human *UBC*+) in the tumor microenvironment using a combination of gene expression markers and spatial location (Figs. S8A-C). *Bglap*^+^ osteoblasts localized to bone surfaces and were decreased in the MM group but significantly increased in both naïve mice and mice bearing MM tumors treated with Scl-ab (Figs. 6D-E). *Limch1*^+^ osteo-CAR cells were found primarily on bone surfaces and a few in the bone marrow (Figs. 6F-G). Similar to osteoblasts, osteo-CAR cells on bone surfaces were decreased by MM tumors and increased by Scl-ab treatment. Scl-ab only modestly increased *Limch1*^+^ cells located in the bone marrow (Fig. S6D). *Cxcl12*^+^ adipo-CAR cells were preferentially located in the bone marrow and unaffected by MM tumors or Scl-ab (Figs. 6H-I). Bones from mice with MM tumors had a higher prevalence of empty osteocyte lacunae compared to control mice, an observation that was not affected by Scl-ab treatment (Fig. S8E). Together, our findings from scRNAseq analysis and spatial quantification highlight osteoblasts and osteo-CAR cells as the osteoblastic populations most susceptible to inhibition by MM cells and activation by Scl-ab.

### Scl-ab improves bone health in patients with MM in remission

**Fig. 7:**
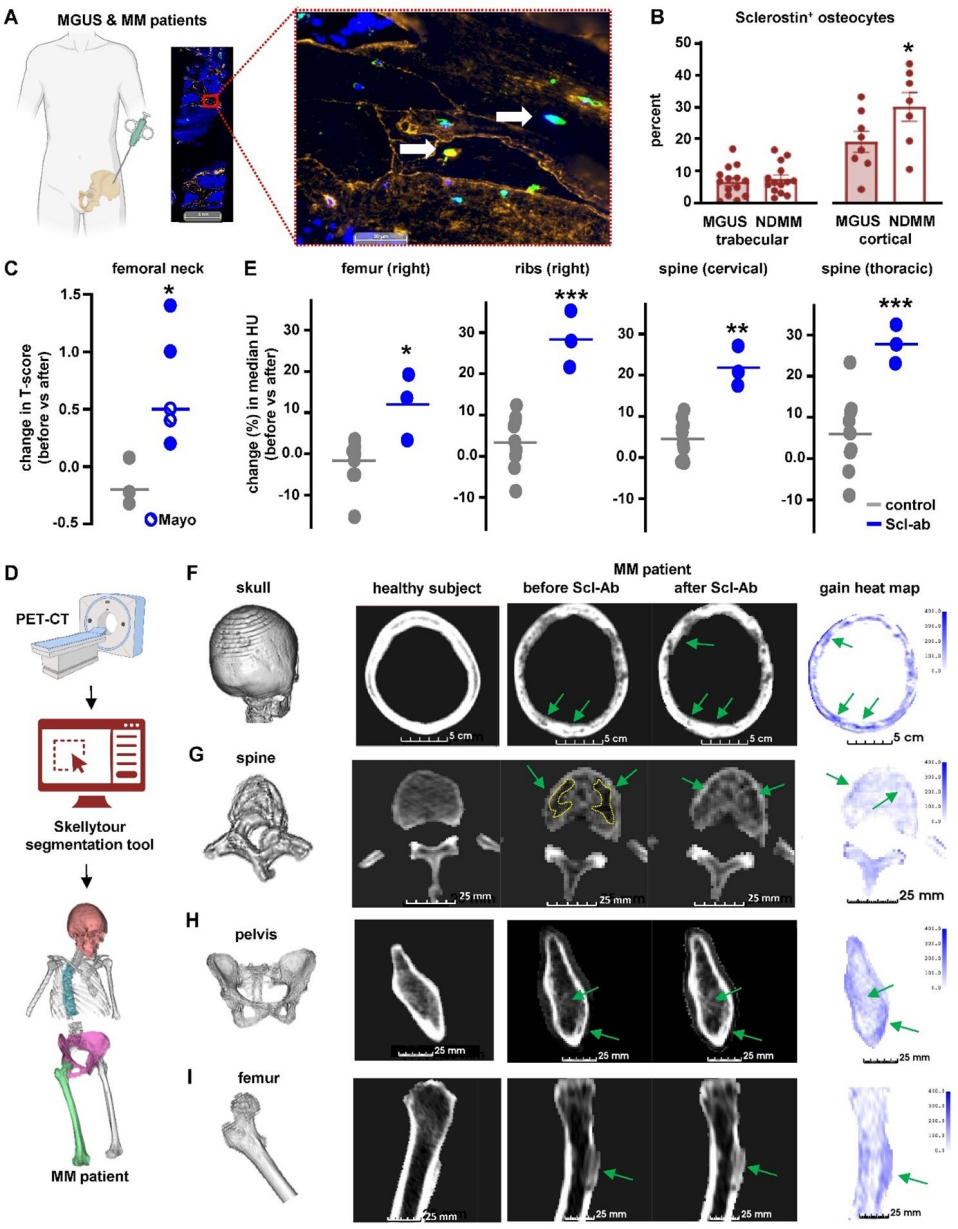
Anti-sclerostin-antibody effects on bone health in MM patients in remission. (**A**) Representative images of a bone biopsy from a patient with newly diagnosed MM and enlargement of the tissue section showing osteocytes positive for sclerostin (green), bone matrix protein osteopontin (yellow), and Hoechst-stained nuclei (blue). White arrows denote sclerostin^+^ osteocytes. Scale bars, 2 mm and 50 μm, respectively. (**B**) Percent of sclerostin^+^ osteocytes in trabecular or cortical bone from patients with monoclonal gammopathy of undetermined significance (MGUS) or newly diagnosed MM. *n* = 12–14 patients/group. *p < 0.05 vs. control by Student’s t-test. (**C**) Changes in femoral neck bone mineral density (BMD) T-scores during follow-up of patients with MM in remission treated with/without anti-sclerostin-antibody (Scl-ab). *n* = 3–5 patients/group. Each dot represents an independent patient. (**D**) Experimental design to calculate radiodensity from PET-CT scans of patients with MM in remission treated with/without Scl-ab using the segmentation tool Skellytour. (**E**) Percent change in median radiodensity Hounsfield Units (HU) in femur, ribs, and spine from patients with MM in remission treated with/without Scl-ab. *n* = 3–10 patients/group. *p < 0.05; **p < 0.01; ***p < 0.001 vs. control. Each dot represents an independent patient. Representative images of a healthy subject, a patient with MM in remission treated with Scl-ab (before and after), and a BMD gain heat map (before vs. after) showing improvements in bone health and bone repair in the skull (**F**), spine (**G**), pelvis (**H**), and femur (**I**). Scale bars, 5 cm (**F**) and 25 mm (**G**-**I**). “Before” refers to PET-CT scans taken prior to the initiation of Scl-ab treatment, and “after” refers to those taken after the completion of the treatment course

Prior work has demonstrated that MM cells reduce the number of viable osteocytes in both patients and animal models [10, 23, 24], while also stimulating sclerostin production by osteocytes, though this has only been reported in preclinical models [10, 24]. Sclerostin serum levels are increased in patients with MM [11], but its production at the tissue levels has not been explored in MM patients. To address this gap, we examined sclerostin production in bone biopsies from patients with MM or MGUS, a precursor stage of the MM disease. Patients with MM exhibited a higher prevalence of cortical osteocytes expressing sclerostin compared to patients with MGUS (Figs. 7A-B). We lastly assessed whether Scl-ab could improve bone health in MM patients. Towards this end, we retrospectively analyzed the effects of Scl-ab on bone mineral density (BMD) and tumor burden in 5 patients with MM who achieved complete remission. Table S2 summarizes the patients’ characteristics. Available positron emission tomography-computed tomography (PET-CT) and dual-energy X-ray absorptiometry (DXA) scans, along with serum paraprotein measurements, were used to assess bone health and tumor status. Patients with MM in remission who did not receive Scl-ab serve as controls. Notably, none of the patients receiving Scl-ab relapsed during treatment, as confirmed by PET-CT imaging and undetectable levels of M-protein (Table S2 and Figs. S9A-B). Due to the retrospective nature of the study, we did not match cohorts of patients for the presence of osteoporosis, and MM patients receiving Scl-ab exhibited the lowest T-scores at baseline, before Scl-ab administration. Thus, we compared T-score changes within each individual, rather than absolute T-scores, for each group. All patients treated with Scl-ab exhibited an increase in BMD at the femoral neck, with T-scores rising between 0.3 and 1.4 after treatment (Fig. 7C). To expand our analysis to other bone sites, we used AI-assisted segmentation [25] to analyze bone density from CT scans taken before and after Scl-ab therapy (Fig. 7D). This analysis revealed bone gain at multiple sites, including the femur, ribs, and spine (Fig. 7E and Fig. S9C). These gains were greater than those seen in controls, despite all the patients in the control group but one receiving anti-resorptive therapy (Fig. 7C, Fig. S9C, Table S2). Moreover, significant repair of lytic lesions was observed in the skulls, vertebrae, pelvis, and femurs of MM patients receiving Scl-ab therapy (Figs. 7F-I). While these clinical findings strongly suggest that Scl-ab therapy improves bone health in MM patients in remission, this retrospective analysis must be interpreted with caution due to the small sample size and patient and treatment heterogeneity.

## Discussion

In this study, we investigated several previously unknown aspects of reversing aberrant inhibition of Wnt signaling in the MM tumor microenvironment with Scl-ab. First, we demonstrate that neutralizing sclerostin preserves the efficacy of BT-GSI, an anti-tumor therapeutic agent. Second, we show that Scl-ab promotes bone repair and heals lytic lesions and identify specific subpopulations of osteoblastic lineage cells that respond to this treatment. Third, our retrospective analysis, though limited by sample size and patient heterogeneity, suggests that Scl-ab may improve bone health and promote bone repair in patients with MM in remission. Collectively, our findings provide evidence that Scl-Ab could significantly improve bone health in MM patients, offering a compelling rationale for conducting prospective, randomized trials to assess its long-term benefits and effects on disease progression and quality of life.

Wnt signaling activation in MM cells has been shown to support tumor progression and contribute to the pathogenesis of MM [26]. Due to its potential to stimulate tumor growth, caution has been exercised regarding the use of therapeutic agents that activate Wnt in the context of cancer. However, previous studies by us and others showed that Scl-ab does not affect tumor growth in animal models of MM [10, 12, 13]. In this study, we confirmed that Scl-ab does not activate a transcriptional program in MM cells that leads to enhanced proliferation. However, our analysis showed other transcriptional changes induced by Scl-ab in MM cells. It is well established that osteoblastic lineage cells can influence MM cell transcriptome [27], suggesting that the transcriptomic effects of Scl-ab in MM cells may involve indirect mechanisms through interactions with osteoblastic cells of the tumor niche. Yet, MM cells express the sclerostin co-receptor Lrp4 at the transcriptional level and thus could directly respond to sclerostin. We also showed that Wnt activation via Scl-ab does not promote MM growth in ex vivo organ cultures derived from human bone. Additionally, we observed that Scl-Ab did not cause disease relapse during the treatment course in five patients with MM who were in remission. However, this observation must be interpreted with caution due to the small sample size and the lack of extended follow-up, which limits our ability to exclude the possibility of delayed effects on disease progression. Importantly, Scl-ab does not interfere with the efficacy of BT-GSI or Carfilzomib, as shown before [13], in animal models. These results suggest that Scl-ab may be safely combined with other therapies, although further investigation into potential drug interactions is necessary.

The mechanisms leading to osteoblast suppression in MM are complex. Previous work has primarily focused on the effects of MM cells on mesenchymal stem cells and osteoblasts in vitro [28]. Our scRNAseq study is among the first to investigate the impact of MM cells on various primary osteoblastic cells in vivo. We show that MM cells eliminate and reprogram osteoblastic cells, particularly osteoblasts and osteo-CAR cells, which exhibit Wnt signaling inhibition and suppression of genes involved in osteoblast differentiation and proliferation. Osteo-CAR cells have been recently proposed as a local source of osteoblast progenitors [19] based on their close association with osteoblasts on the bone surface [19] and the ability of CAR cells to differentiate into osteoblasts [29, 30]. We also identified a population of osteoblasts, osteoblast_2, that persisted in MM-bearing bones and expressed low levels of Lrp4, suggesting that these cells may be less susceptible to sclerostin-Lrp4 suppression. Further studies are warranted to investigate the characteristics of this population and its role in the progression of MM and bone disease. Lastly, we did not observe significant changes in the number of adipo-CAR cells, but this population displayed transcriptional reprogramming towards pro-angiogenesis.

Our study is the first to show the capacity of Scl-ab to not only increase bone mass, as previously shown [10, 12, 13], but also to promote the healing of osteolytic focal lesions in both animal models and patients. Notably, Scl-ab activates Wnt signaling and stimulates osteoblast differentiation and proliferation programs in both osteoblast and osteo-CAR cells in naïve mice and restores these populations to control levels in mice bearing MM tumors, suggesting that sclerostin influences the function of these populations. It is important to note that our analysis was limited to osteoblastic lineage cells and did not include other bone marrow cell populations, such as immune cells, which may play a role in mediating the response to or could be influenced by Scl-ab. Moreover, whether the bone repair effects of Scl-ab are mediated through actions on osteoblasts or osteo-CARs, recovery of residual diseased osteoblasts (i.e., osteoblast_2 population), activation of lining or mesenchymal stem cells as shown in naïve mice [31, 32], or a combination of these processes, remains uncertain and requires further study. Loss of Wnt/beta-catenin signaling is known to shift the cell fate of osteoprogenitors from osteoblasts to adipocytes [33]. In our study, Scl-ab did not affect the number of adipo-CAR cells, which can differentiate into adipocytes [22]. One possible explanation for this observation is the differential expression of Lrp4, which is significantly lower in adipo-CAR cells (Fig. 5D). This reduced Lrp4 expression could make adipo-CAR cells less susceptible to Wnt signaling inhibition via sclerostin-Lrp4 signaling (Figs. 6B-C). Additionally, it is possible that in MM, where marrow adipocytes are increased [34], MM cells may influence adipo-CAR cells through Wnt-independent pathways, which could explain why the Adipo-CAR population remains unaffected by Scl-ab treatment.

Co-administration of BHQ880, a neutralizing antibody against Dkk1, with anti-MM and anti-resorptive therapy, resulted in only modest improvements in bone health in a cohort of MM patients with relapsed/refractory disease [35]. In our retrospective clinical study, romosozumab significantly improved bone health and repaired osteolytic lesions in patients with MM in remission (5/5-100%), with no evidence of disease relapse during the course of the treatment. Notably, patients treated with romosozumab experienced a greater increase in bone mass compared to the control group, even though the controls were receiving anti-resorptive therapy. These improvements are noteworthy, particularly given the severity of osteoporosis in these patients. Prior treatment with anti-resorptive has been shown to attenuate the bone anabolic effects of romosozumab [36, 37]. Therefore, it is unlikely that the gains seen in the two patients from the Mayo Clinic were due to prior treatment with bisphosphonates. Romosozumab carries a black box warning for potential myocardial infarction or stroke in the preceding year. No cardiovascular events were reported in patients receiving romosozumab during this study. Although these results are promising, they should be interpreted with caution due to the limitations inherent in the retrospective design of the study, as well as the small sample size, lack of matching between cohorts, and patient and treatment heterogeneity. Moreover, the follow-up duration was insufficient to evaluate long-term fracture incidence and progression-free survival. Larger, prospective, randomized trials are needed to assess the long-term effects of romosozumab on skeletal-related events, quality of life in patients with MM, and disease progression. Of note, the beneficial skeletal impact of Scl-ab could be enhanced by the co-administration (as opposed to sequential treatment) of bisphosphonates and is not influenced by anti-cancer agents or chemotherapy, as demonstrated in both this and previous preclinical studies [12, 13]. These findings suggest that not only patients in remission but also patients at other stages of the disease, including those with MGUS who exhibit increased fracture risk [38] or newly diagnosed MM patients, could benefit from romosozumab therapy. Lastly, bone remineralization and repair in MM remain an underexplored area due to the lack of proper analytic tools, and the regression of lytic lesions has only been reported in subsets of MM patients [39, 40]. Our work also highlights the potential of AI-assisted imaging segmentation tools for evaluating bone health using CT scans, enabling consistent comparisons of the same anatomical site over time in MM patients.

Proteasome inhibitors have bone anabolic effects in certain populations of MM patients, although these benefits may be transient [40, 41, 42]. These agents stimulate osteoblast differentiation and bone-forming activity by preventing the proteasomal degradation of key osteogenic transcription factors, such as Runx2, thereby increasing the expression of bone-related genes, including osteocalcin and alkaline phosphatase [43, 44]. Additionally, proteasome inhibitors promote osteocyte survival, particularly in the setting of MM and dexamethasone-induced apoptosis [45]. They have also been shown to downregulate Dkk1 expression, which can help restore Wnt signaling and further support osteoblast function [46]. These mechanisms of action differ from those of Scl-ab, which rely exclusively on the activation of Wnt signaling to enhance osteoblast and osteocyte survival and function [47, 48]. Interestingly, in the two patients receiving VRd or lenalidomide maintenance therapy alongside romosozumab, we observed greater improvements in bone mineral density, suggesting potential additive beneficial effects on bone due to the anabolic and anti-resorptive properties of proteasome inhibitors and lenalidomide [40, 49]. This observation underscores the need for studies to determine the most effective combinatorial regimens for MM patients, particularly with regard to their effects on both bone health and tumor progression. Moreover, this study highlights the potential for exploring other bone anabolic agents in clinical practice, such as intermittent PTH or Dkk1-ab, which have been shown to exhibit bone anabolic potential in the context of MM [50, 51, 52, 53].

## Conclusions

In conclusion, our findings provide new evidence supporting the use of Scl-ab to repair damaged bone and counteract the deleterious effects of MM in the skeleton. The combination of Scl-ab with anti-cancer therapeutics offers a unique combination of anti-cancer and bone anabolic effects, which are often not observed with current therapeutic regimens. Our data provide a strong and compelling rationale for conducting larger and prospective clinical trials in MM patients using romosozumab as an adjuvant.

## Supplementary Information

Below is the link to the electronic supplementary material.

Supplementary Material 1

## Data Availability

The sc-RNA-seq and bulk RNAseq data generated in this study were deposited in the NCBI SRA database under the Bioproject PRJNA1210176 and PRJNA1211159. All other raw data are available upon request from the corresponding author.

## Abbreviations

MM: Multiple myeloma
Scl-ab: Neutralizing antibody against sclerostin
CAR: Cxcl12-abundant-reticular cell
BT-GSI: Bone-targeted Notch inhibitor
GSI: Gamma secretase inhibitor
PET-CT: Positron emission tomography-computed tomography scan
DXA: Dual-energy X-ray absorptiometry
