# Supplementary material for: Healing of lytic lesions and restoration of bone health in multiple myeloma through sclerostin inhibition
Source: Exp Hematol Oncol. 2025 Aug 22;14:108. doi: 10.1186/s40164-025-00699-4 (PMC12372396; doi:10.1186/s40164-025-00699-4)
Supplement: Supplementary file 1 — Supplementary Material 1 [file 40164_2025_699_MOESM1_ESM.pdf]

Supplementary Materials for

Healing of Lytic Lesions and Restoration of Bone Health in Multiple Myeloma Through Sclerostin Inhibition

Hayley M. Sabol, Aric Anloague, Japneet Kaur, Cecile Bustamante-Gomez, Sharmin Khan, Bethany C. Paxton, Mattie R. Nester, Jillian Hackney, Marta Diaz-delCastillo, Daniel Mann, Jeffrey B. Stambough, C. Lowry Barnes, Elena Ambrogini, Alison Frontier, Frank H. Ebetino, Syed Naqvi, Frits van Rhee, Christopher Wardell, Matthew T. Drake, Intawat Nookaew, Carolina Schinke, Maurizio Zangari, and Jesus Delgado-Calle.

*Corresponding author. Email: JDelgadocalle@uams.edu

The PDF file includes:

Materials and Methods

Fig. S1. Effects of Scl-ab, BT-GSI, and combo therapy on tumor burden in bone and spleen weight.

Fig. S2. Effects of BT-GSI, Scl-ab, and combo therapy on the transcriptome of MM cells.

Fig. S3. Go Term and Notch and proliferative pathways analyses of the effects of BT-GSI, Scl-ab, and combo therapy on MM cells.

Fig. S4. Cancellous bone repair and bone formation parameters in immunocompetent mice treated with Scl-ab.

Fig. S5. Cancellous and cortical microarchitectural parameters in immunodeficient mice treated with Scl-ab.

Fig. S6. Impact of combo treatment on human bones bearing human MM tumors cultured ex vivo.

Fig. S7. Identification of osteoblastic cell populations in the scRNAseq dataset.

Fig. S8. Histological analysis of osteoblastic populations in bones from naïve mice or MM-bearing mice treated with Scl-ab.

Fig. S9. Tumor and bone analysis in PET-CT scans from MM patients.

Table S1. Toxicology studies.

Table S2. Patients' demographics and study characteristics.

References

Other Supplementary Material for this manuscript includes the following:

Data file S1. Gene expression and GO term analysis in BTGSI_vs_veh_MM cells.

Data file S2.Gene expression and GO term analysis in BTGSI + Scl-ab_vs_veh_MM cells.
Data file S3.Gene expression and GO term analysis in Scl-ab_vs_veh_MM cells.
Data file S4.Gene expression and GO term analysis in MM_vs_N_Adipo-CAR.
Data file S5.Gene expression and GO term analysis in MM vs_N_Osteoblasts1.
Data file S6.Gene expression and GO term analysis in MM_vs_N_Osteo-CAR.
Data file S7.Gene expression and GO term analysis in MM_vs_N_Osteoblasts2.
Data file S8.Gene expression and GO term analysis in MM_vs_N_Osteocytes.
Data file S9.Gene expression and GO term analysis in SclAb_vs_N_Osteoblasts2.
Data file S10.Gene expression and GO term analysis in SclAb_vs_N_Osteo-CAR.
Data file S11.Gene expression and GO term analysis in SclAb_vs_N_Osteoblast1.
Data file S12.Gene expression and GO term analysis in SclAb_vs_N_Osteocytes.
Data file S13.Gene expression and GO term analysis in SclAb_vs_N_Adipo-CAR.

Materials and Methods

Reagents. Murine 5TGM1 MM cells (RRID: CVCL_VI66) and human OPM2 MM (RRID: CVCL_1625) cells were provided by Dr. Oyajobi (University of Texas at San Antonio, TX, USA) and Dr. Roodman (Indiana University). MM cell lines were cultured as previously described (1, 2). RPMI 1640 media, fetal bovine serum, Normocin, Plasmocin, antibiotics (penicillin/streptomycin), TriZol, and DiD cell trackers (Cat#V-22887) were purchased from Invitrogen Life Technologies (Grand Island, NY, USA). Puromycin (Cat#58-58-2) and Blasticidin (Cat# ant-bl) were purchased from InvivoGen (San Diego, CA, USA). Alizarin red (A3882-5G) and Calcein (Cat # C0875-5G) were purchased from Sigma-Aldrich (St. Louis, MO, USA). Anti-sclerostin antibody (romosozumab or Evenity) was purchased from the UAMS pharmacy, and melphalan (Cat# M2011) was purchased from Sigma-Aldrich (St. Louis, MO, USA). Coelenterazine (Cat# 303-500) was purchased from Nanolight Technology (Pinetop, AZ, USA).

Toxicity studies. For the assessment of systemic (organ) functional toxicity, blood was collected from immunocompetent mice injected intratibially with 5TGM1 MM cells or saline and treated with vehicle, Scl-ab, BT-GSI, or a combination of the two for 4 weeks. Measurement of 14 blood parameters indicative of organ function was performed using VetScan VS2 instrument equipped with the Comprehensive Diagnosis Kit (Abaxis, Union City, CA), conducted by the Cellular and Molecular Toxicology Core (University of Arkansas for Medical Sciences, AR, USA).

Flow cytometry. The percent of mCherry+ MM cells in the bone marrow of bones cultures ex vivo was assessed using flow cytometry (LSR Fortessa; BD Biosciences, San Jose, CA, USA). The bone marrow from bones was flushed out, red blood cells were lysed, and the remaining cells were stained with DAPI. The samples were analyzed using Flowjo (Ashland, OR, USA).

Bioluminescence. Luciferase activity was assessed in explanted bones, including the femur, tibia, and L5. Bones were incubated in coelenterazine for 5 minutes and then imaged using the IVIS Lumina XRMS system (Waltham, MA, USA), as previously described (3).

RT-qPCR. Total RNA was isolated from the bone tissues using Trizol and converted to cDNA (Invitrogen Life Technologies) according to the manufacturer's directions. Gene expression was quantified using quantitative real-time PCR (qPCR) with TaqMan assays from Applied Biosystems (Foster City, CA, USA), following the manufacturer's instructions. Gene expression levels were calculated using the comparative threshold (CT) method and were normalized to the housekeeping gene GAPDH.

microCT. MicroCT imaging was performed in live mice or *ex vivo* explanted bones using a vivaCT 80 (Scanco Medical AG, Switzerland). Bones were aligned to the proximal axis, and analysis of the cancellous bone was performed in the tibia, femur, or L5 vertebrae in an area 20 μm below the growth plate, using a 10 μm resolution**.** Lytic lesions were visualized in 3D reconstructions and quantified in the cortical bone between the growth plate and the midshaft of the bone.

Enzyme-linked immunoassays (ELISA). The levels of the tumor biomarkers human lambda (Bethyl Laboratories, Montgomery, TX, USA; Cat#E88-116) or IgG2B (Invitrogen, Grand Island, NY, USA; Cat#88-50430-88), paraproteins produced by OPM2 and 5TGM1 cells, respectively, were used to determine tumor growth/burden *in vivo* (serum) and *ex vivo* (conditioned media). The bone resorption biomarker C-telopeptide of type 1 collagen (CTX) (murine: Immunodiagnosticsystems, Boldon, UK; Cat#AC-06F1; human: Immunodiagnosticsystems, Cat#AC-02F1) and bone formation marker propeptide of type 1 collagen (P1NP) (murine: Immunodiagnosticsystems, Cat#AC-33F1; human: Biomatik, Wilmington, Delaware; EKU10200) were analyzed in serum (*in vivo*) or conditioned media (*ex vivo*).

Bone Histomorphometry. Static and dynamic bone histomorphometric analyses were performed using the OsteoMeasure High-Resolution Digital Video System (OsteoMetrics, Decatur, GA, US), as previously described (4). Analyses were conducted in the cancellous bone of vertebrae or tibiae, starting 200 μm below the growth plate (1, 2).

RNAseq and Bioinformatic analyses. For scRNAseq, cells per condition were encapsulated using a Chromium Controller (10X Genomics, Pleasanton, CA, US), and libraries were constructed using a Chromium Single Cell 3' Reagent Kit (10X Genomics) by the UAMS Genomics Core or Flow Cytometry Core. Libraries were sequenced using an Illumina NovaSeq 600 machine to generate fastq files, as shown before. Cells per condition were encapsulated using a Chromium Controller (10X Genomics, Pleasanton, CA, US), and libraries were constructed using a Chromium Single Cell 3' Reagent Kit (10X Genomics) by the UAMS Genomics Core or Flow Cytometry Core. Libraries were sequenced using an Illumina NovaSeq 600 machine to generate fastq files, as shown before (3). For bulk RNA sequencing, the libraries were sequenced on Illumina NovaSeq platforms to generate paired-end reads of 150 bp in length. Bulk RNAseq analysis was performed follwoing our bioinformatic pipeline (5). The fastq files were mapped to the mouse reference genome mm10 using STAR (6). The gene count table was generated using BEDTools 2 (7) and imported into the R suite software environment. The data was normalized using Voom with the quantitative quality weights method (8). The differential gene expression analysis was performed using the moderate Student's t-test (9) to compare the different groups versus the control group, and the p-values were further adjusted for multiple testing using the Benjamini-Hochberg method. For scRNAseq analysis, the fastq file alignments were performed using the mouse reference genome mm10, preprocessed using CellRanger v7, and then imported into the R suite software environment using the Seurat package v5 (10-12). Quality control

protocols were applied to remove outlier barcodes based on the depth, number of genes, and proportion of mitochondrial genes. The harmonization/integration of different samples was performed using the canonical correlation analysis (CCA) method (11). UMAP was employed for dimensional reduction and visualization of high-quality cells. Subpopulation identification and clustering were performed using the Louvain algorithm with multilevel refinement (13). Gene-specific markers of individual clusters were identified using the FindMarkersAll function with the MAST algorithm for cell type identification (14), following the cell type annotation as previously published (14). Functional pathway enrichment analysis was performed using PIANO software (15). Z-score was used to calculate statistical differences.

RNAscope. L4-6 spines were processed for paraffin sectioning by first fixing in Millonig's 10 % buffered formalin for 40 h at 4 °C. Bones were then decalcified in 14 % EDTA for one week, after which they were dehydrated into 100% ethanol and embedded in paraffin for sectioning. All aqueous solutions were prepared with DEPC-treated water. RNA *in situ* hybridization was performed using the RNAscope 2.5 HD detection reagent RED (cat. no. 322360) kit, following the manufacturer's instructions (Advanced Cell Diagnostics, Newark, CA). In brief, eight-micron paraffin sections were incubated at 60 °C for 60 min, deparaffinized, and pretreated with 3% Hydrogen Peroxide for 15 min at RT. L4-6 lumbar spine serial sections were incubated with human UBC (cat.#310041), murine Bgalp (cat. #478941), Limch1 (cat. #591801), or Cxcl12 (cat. #422711) probes for 2h at 40 °C. The signal was then detected at room temperature for 10 min using RNAscope detection reagent RED (cat. #322360), counterstained with hematoxylin and ammonia water, dehydrated at 60 °C for 20 min, and mounted with VectaMount permanent mounting medium (Vector Laboratories). Analyses were performed in the cortical or cancellous bone of vertebrae, starting 200 μm below the growth plate. Empty osteocyte lacunae, Bglap and

Limch1 positive cells on the bone surface, and Cxcl12 or Limch1 positive cells in the bone marrow (five random fields per sample) were quantified using Osteomeasure software at 40x magnification.

PET-CT AI-assisted bone segmentation. CT scans were segmented using Skellytour (16), producing 16 bone labels. Data were analyzed in R (R Core Team, 2024; version 4.4.1). For each CT scan segmentation, we extracted the Hounsfield Units (HU) for bone sites of interest. We then took the median value for each bone and calculated the percentage difference between the first and last available scans. To assess the effect of Scl-ab therapy on bone density, we used the package "lme4" to fit a mixed-effects linear regression (Bates et al., 2015; version 1.1-35.5), using the percentage difference in the median HU as the outcome variable and including the treatment group as a fixed effect. Due to the small sample size, we first fit a model to assess the overall effect of Scl-ab therapy on bone density. We included the bone site as a random effect, allowing for both varying intercepts and varying slopes (by treatment group), and included the subject ID as a random effect. To make a full-null comparison, we fit a reduced model in which the grouping variable (treatment or control) was excluded. After running a full-null comparison, we used the `emmeans` package to obtain the estimated marginal mean difference between the treatment and control groups (Lenth, 2024; version 1.10.4). We used the Kenward-Roger approximation to estimate degrees of freedom (17). To account for non-uniform effects, we also selected a subset of bones (femurs, ribs, cervical spine, thoracic spine, and lumbar spine) and fitted a second mixed-effects model, where we included the interaction between bone site and treatment group as a fixed effect and patient ID as a random effect. Likewise, we also tested the model against a reduced model where the grouping variable and the interaction were removed. We used the `emmeans` package to obtain the estimated marginal mean difference between the treatment and control

groups at each bone site. We utilized the Kenward-Roger approximation degree of freedom estimation and the Holm-Bonferroni method to correct for multiple testing correction (18). We used the R package `DHARMa` (Hartig, 2022; version 0.4.6) for model diagnostics. For data visualization, we used 3D Slicer (Fedorov et al., 2012; version 5.6.1) and the R package `ggplot2` (Wickham, 2016; version 3.5.1).

Human samples methods and histological analysis. Bone biopsies (mean age ± standard deviation 71.81 ± 6.60 years) of 3-mm diameter were 4% paraformaldehyde-fixed, decalcified in 0.5 M EDTA, and embedded in paraffin. Tissues were sectioned in 3.5-µm thick slides and deparaffinized in a xylene ethanol gradient, including a 30-minute blocking with 1.5% peroxidase solution. Slides were washed in TBS/Tween buffer and blocked with 5% casein prior to 10 min sequential incubation with avidin and biotin. A biotinylated goat anti-osteopontin antibody was applied (R&D Systems, Cat# BAF1433, RRID:AB_355994) in a 1:50 dilution and incubated o/n at 4°C; visualization was performed with cy3-streptavidin conjugate (Jackson ImmunoResearch Labs Cat# 016-160-084, RRID: AB_2337244). Slides were again washed in TBS/Tween and exposed to sequential 10 min incubations in avidin and biotin prior to the addition of a biotinylated anti-sclerostin antibody (R&D Systems Cat# BAF1406, RRID: AB_2195350) at a 1:100 dilution. Visualization was performed with an Alexa Fluor 488- streptavidin conjugate (Invitrogen Cat# s11223). Finally, slides were counterstained with Hoechst and mounted in fluorescent mounting medium (Prolong Gold). Sections were scanned using an Olympus VS200 slide scanner (Olympus, Japan) and analyzed with AI-assisted histology (IF-FISH v2.1.5, HALO v3.6.4134, Indica Labs). Briefly, trabecular and cortical bone (when available) were manually delineated, and sclerostin+ osteocytes were automatically detected following automatic nuclei segmentation; analyses were conducted by an investigator blinded to study groups.

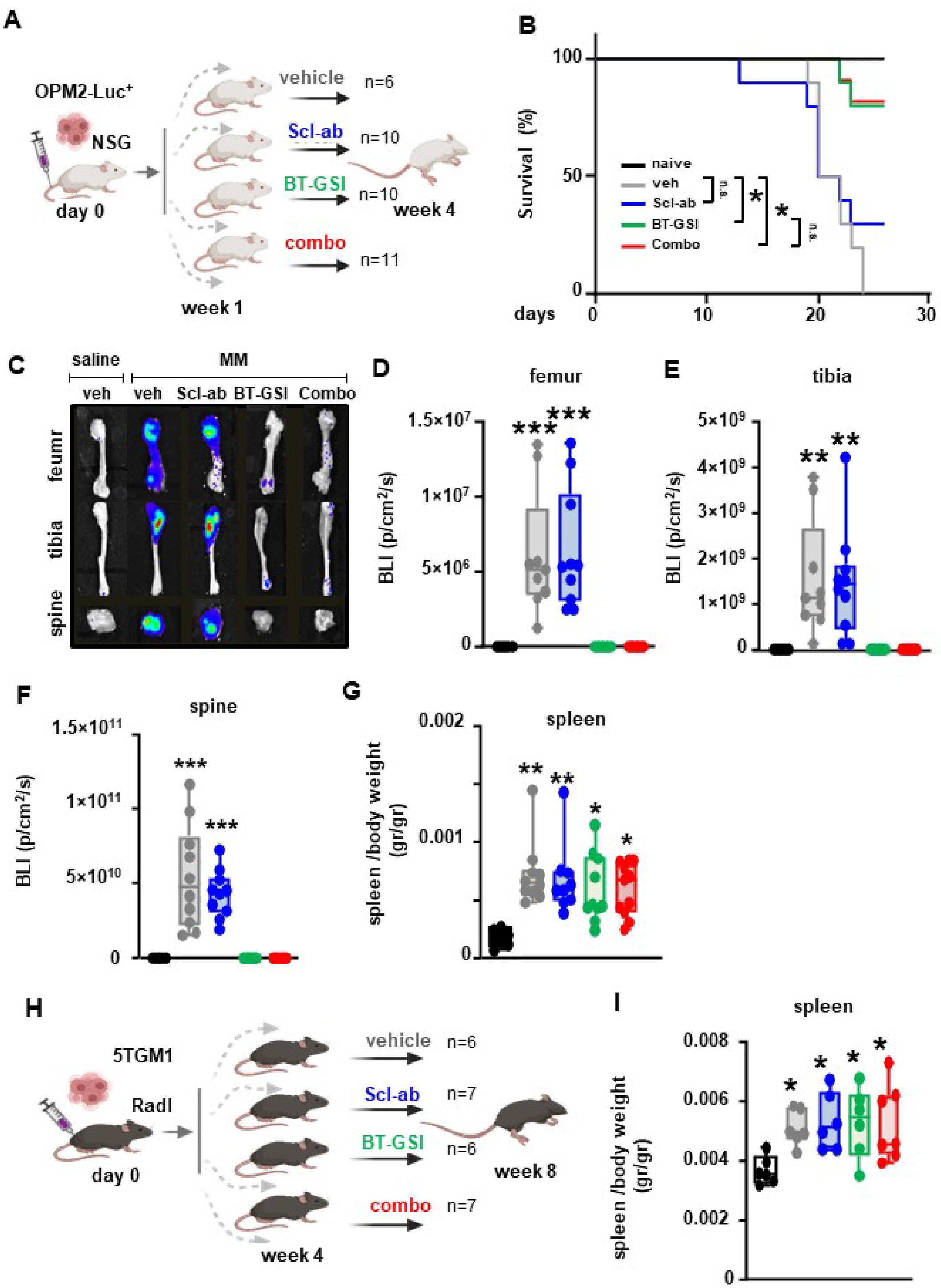

Fig. S1. Effects of Scl-ab, BT-GSI, and combo therapy on tumor burden in bone and spleen weight. (**A**) Human xenograft mouse model experimental design. Effects of Scl-ab, BT-GSI, and combo therapy on survival (**B**), bioluminescence (BLI) of human OPM2-Luc tumors (**C**) in the

femur (**D**), tibia (**E**), and spine (**F**) bones at the endpoint, and spleen weight (**G**). n=6-11 mice/group. (**F**) Immunocompetent mouse model experimental design. Effect of Scl-Ab, BT-GSI, and combo therapy on spleen weight (**I**). n=6-7 mice/group. *p<0.05; **p<0.01; ***p<0.001 vs. vehicle by One-Way ANOVA (D, E, F, G, I) or by a log-rank (Mantel-Cox) test (B). n.s.=non-significant.

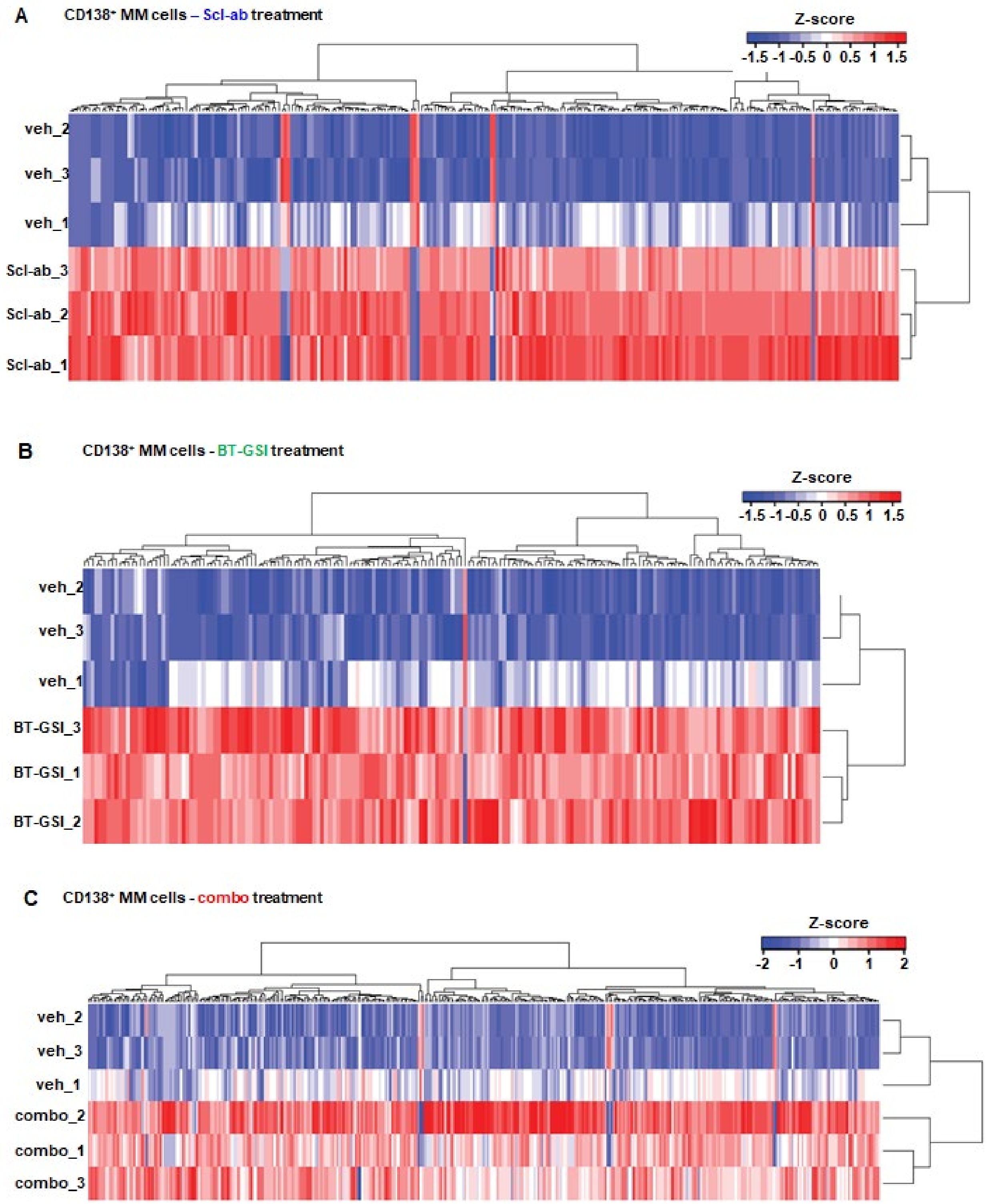

Fig. S2. Effects of BT-GSI, Scl-ab, and combo therapy on the transcriptome of MM cells.

Heatmaps of gene expression by RNA-seq for differentially expressed genes (DEGs) in CD138+

MM cells between the vehicle (veh) and Scl-ab (**A**), veh and BT-GSI (**B**), and veh and combo (**C**), where red is higher expression and blue is lower expression. DEGs were defined as genes with an FDR-adjusted p-value less than 0.05.

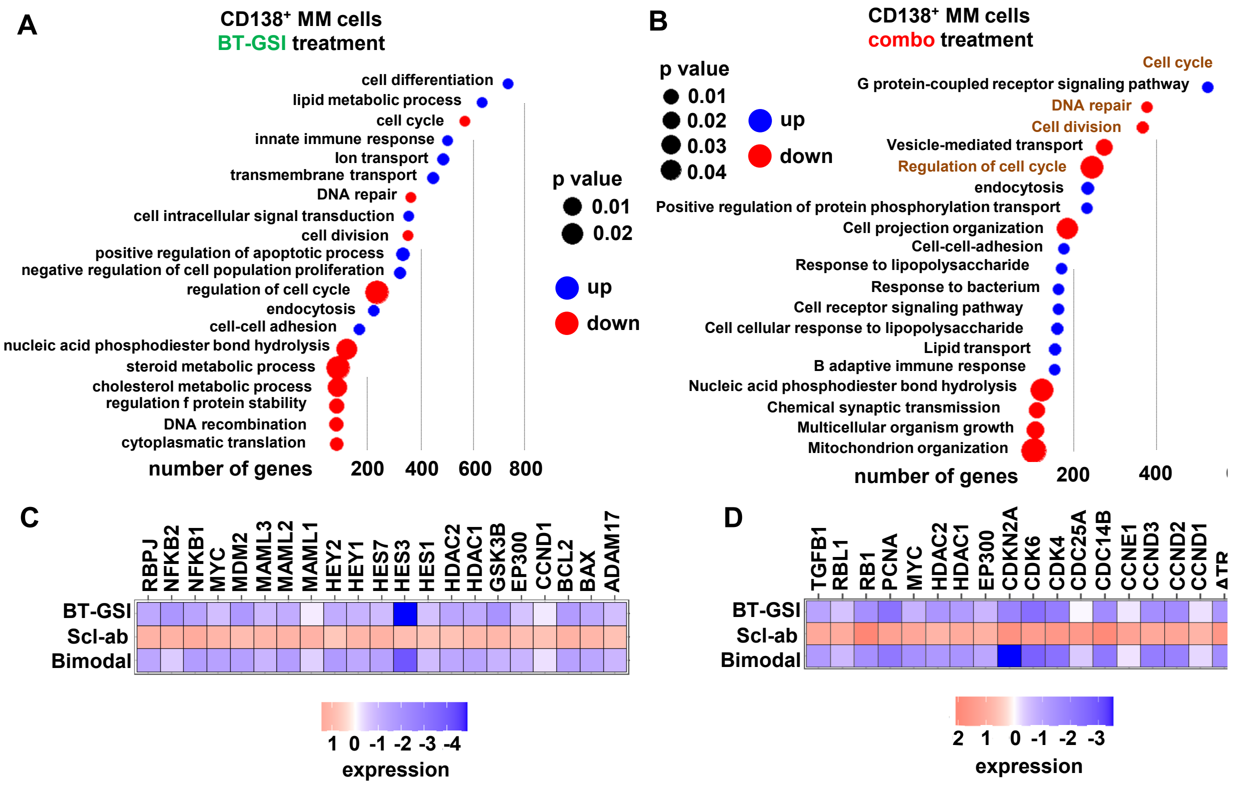

Fig. S3. Transcriptomic analysis of the effects of BT-GSI, Scl-ab, and combo therapy on MM cells. Gene ontology (GO) enrichment analysis in genes differentially expressed in MM cells isolated from mice receiving veh vs. BT-GSI (**A**) or combo (**B**). Bubble size is proportional to the enrichment p-value. Gene expression of Notch target (**C**) and proliferation (**D**) genes from MM cells from mice receiving BT-GSI, Scl-ab, or combo therapy (n=3 mice/group). Color intensity is proportional to the expression of the gene compared to vehicle.

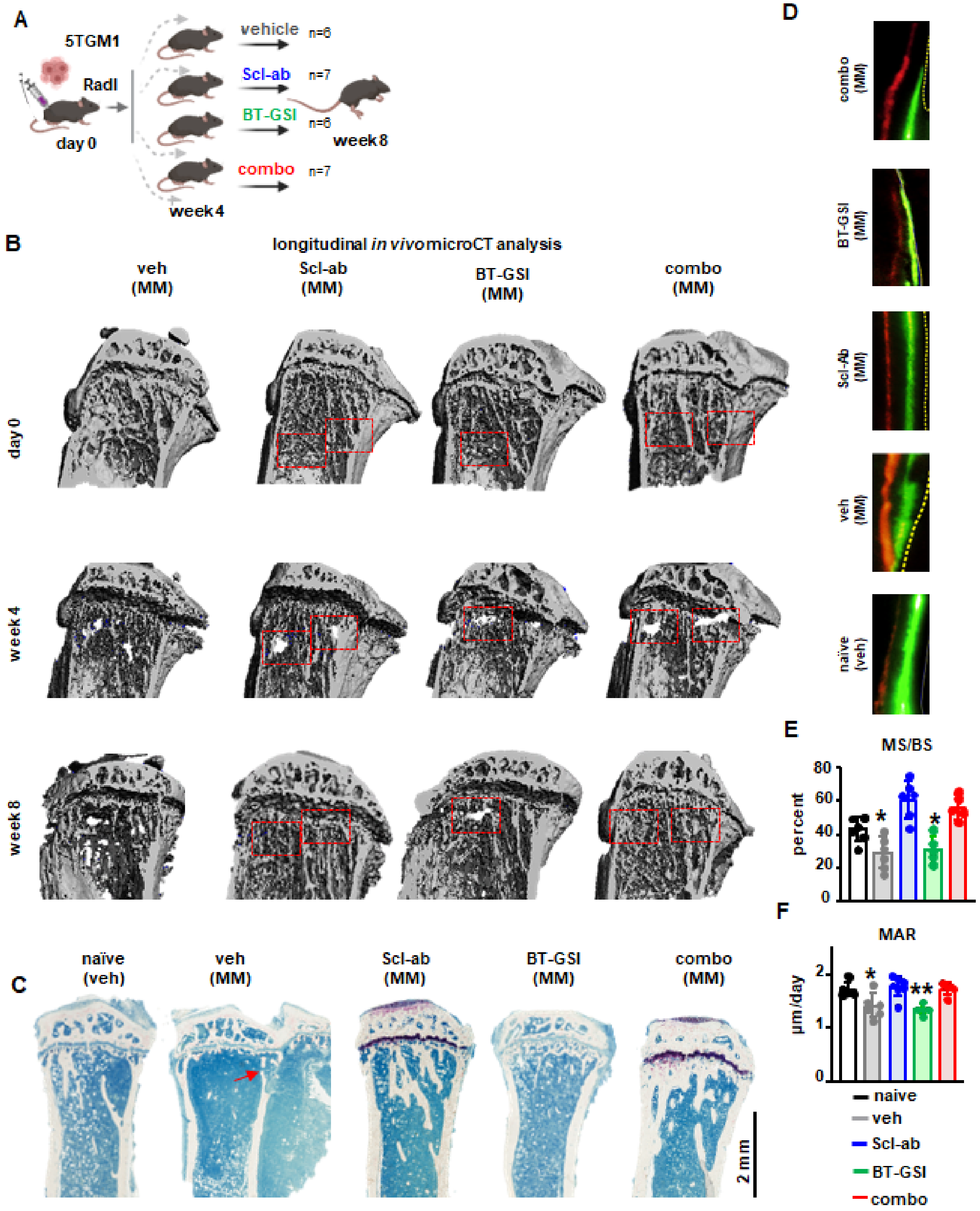
A
5TGM1
RadI
day 0
vehicle n=6
Scl-ab n=7
BT-GSI n=6
combo n=7
week 4
week 8
B
longitudinal in vivo microCT analysis
veh (MM)
Scl-ab (MM)
BT-GSI (MM)
combo (MM)
day 0
week 4
week 8
C
naïve (veh)
veh (MM)
Scl-ab (MM)
BT-GSI (MM)
combo (MM)
2 mm
D
combo (MM)
BT-GSI (MM)
Scl-Ab (MM)
veh (MM)
naïve (veh)
E
MS/BS
percent
F
MAR
µm/day
naive
veh
Scl-ab
BT-GSI
combo

Fig. S4. Cancellous bone repair and bone formation parameters in immunocompetent mice treated with Scl-ab therapy. (**A**) Immunocompetent mouse model experimental design. (**B**) Representative microCT 3D reconstruction longitudinal images of tibiae cancellous bone, (**C**) representative histological sections of tibiae cancellous bone (week 8), (**D**) representative images of calcein (green) and alizarin red (red) labels (scale bar, 50 µm), and (**E-F**) dynamic histomorphometry analysis in cancellous bone in from immunocompetent mice injected intratibially with 5TGM1 MM cells and treated with veh, Scl-ab, BT-GSI or combo therapy for 4 weeks. n=6-7 mice/group. *p<0.05; **p<0.01 vs. naïve by One-Way ANOVA. Red squares indicate areas of repair (B). The red arrow identifies a cortical lytic lesion (C).

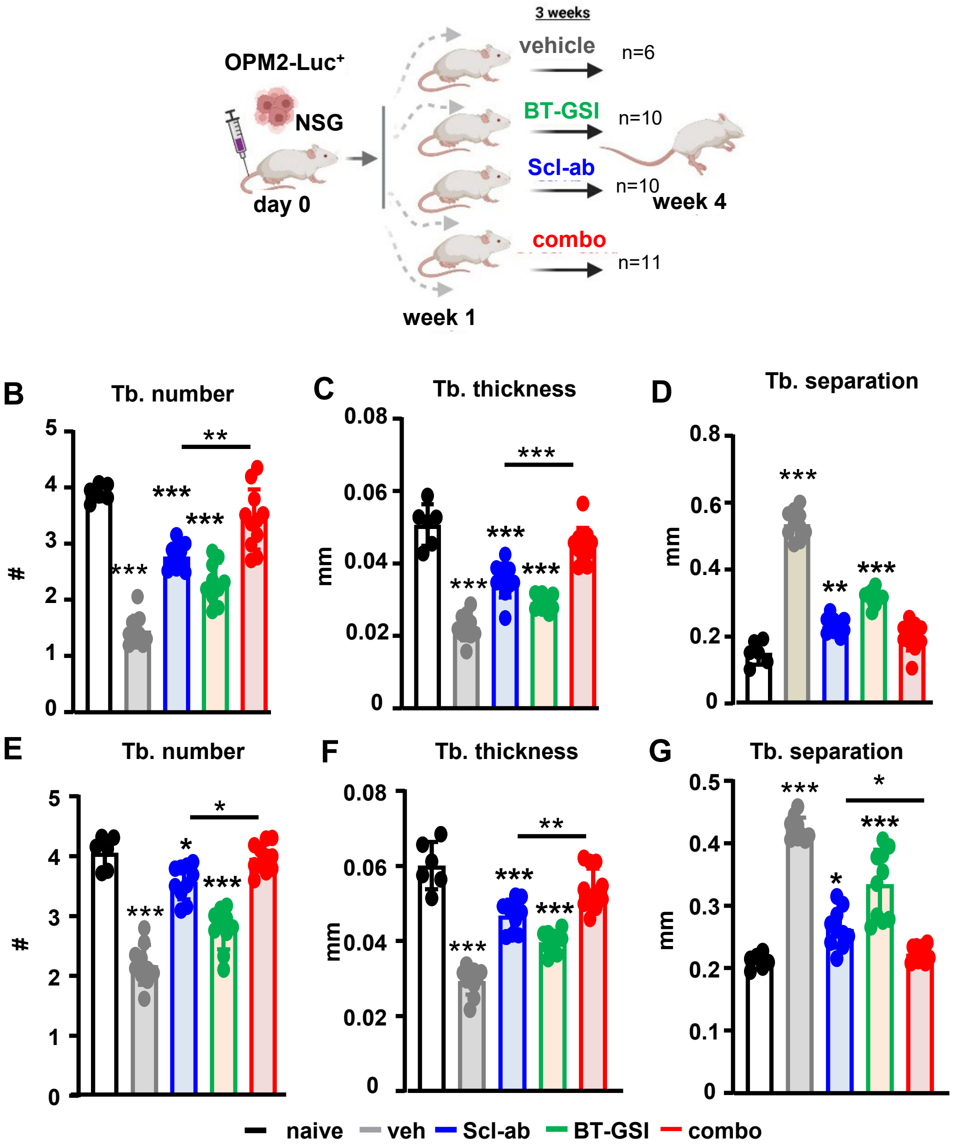

Fig. S5. Cancellous and cortical microarchitectural parameters in immunodeficient mice treated with Scl-ab. (A-D) Immunodeficient mouse model experimental design, femur trabecular (Tb) number, thickness, and separation, and **(E-G)** vertebral trabecular number, thickness, and separation in immunodeficient mice injected with human OPM2 MM cells treated with vehicle, Scl-ab, BT-GSI or combo for 3 weeks. n=6-10 mice/group. *p<0.05; **p<0.01; ***p<0.001 vs. naive by One-Way ANOVA.

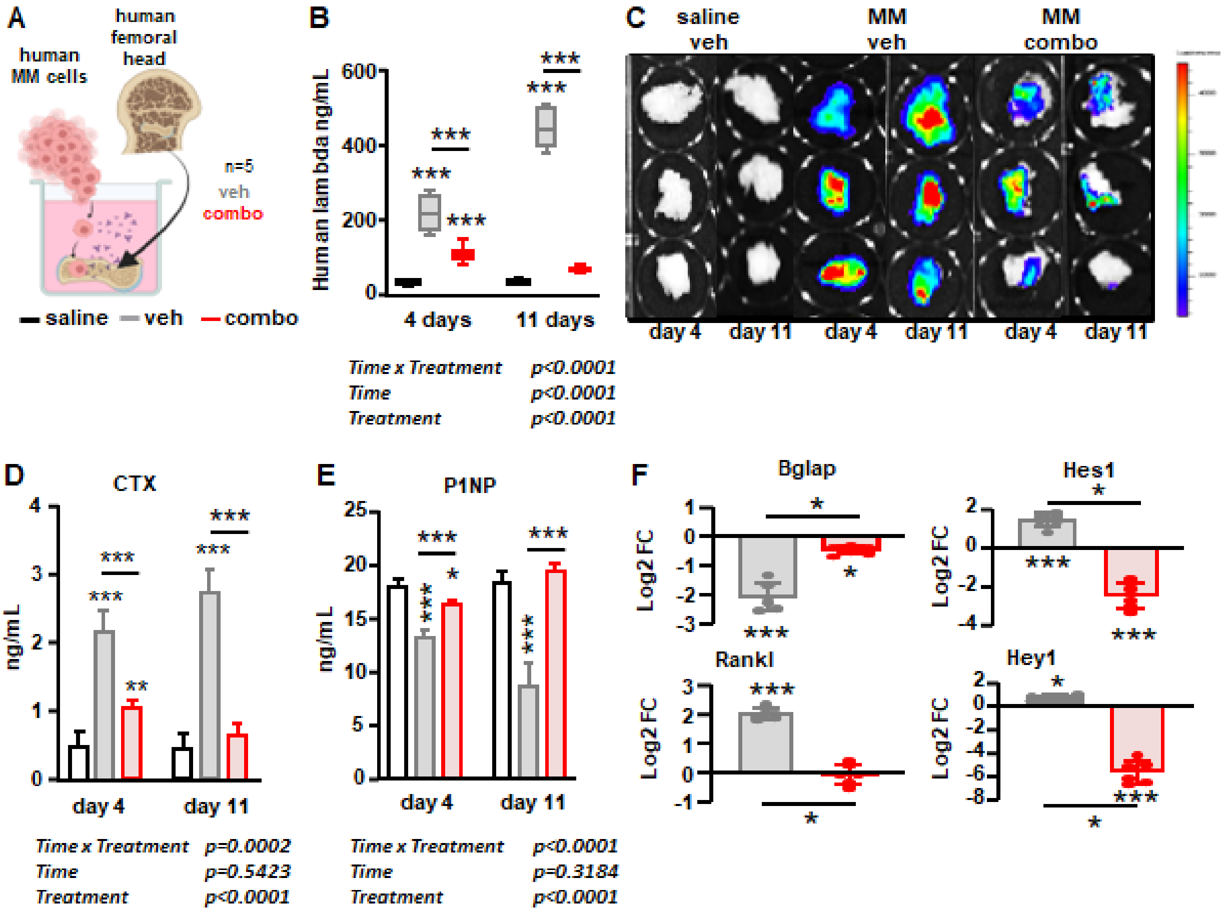

Figure S6. Impact of combo treatment on human bones bearing human MM tumors cultured *ex vivo*. (A) *Ex vivo* bone-MM organ cultures were established using bone fragments from a different control subject and human OPM2-luciferase^{+} MM cells and treated with vehicle or combo therapy. **(B)** Serum human lambda light chain paraprotein levels, **(C)** luciferase activity, **(D)** CTX, and **(E)** P1NP from cultures treated with vehicle or combo therapy after 4 and 11 days. **(F)** Gene expression of *Bglap*, *Rankl*, *Hes1*, and *Hey1* in bones infiltrated with OPM2 MM cells and treated with vehicle or combo. n=5 bones/group. *p<0.05; **p<0.01; ***p<0.001 vs. saline at each time by two-way ANOVA RM (B, D, E), *p<0.05; **p<0.01; ***p<0.001 vs. saline by one-way ANOVA (F).

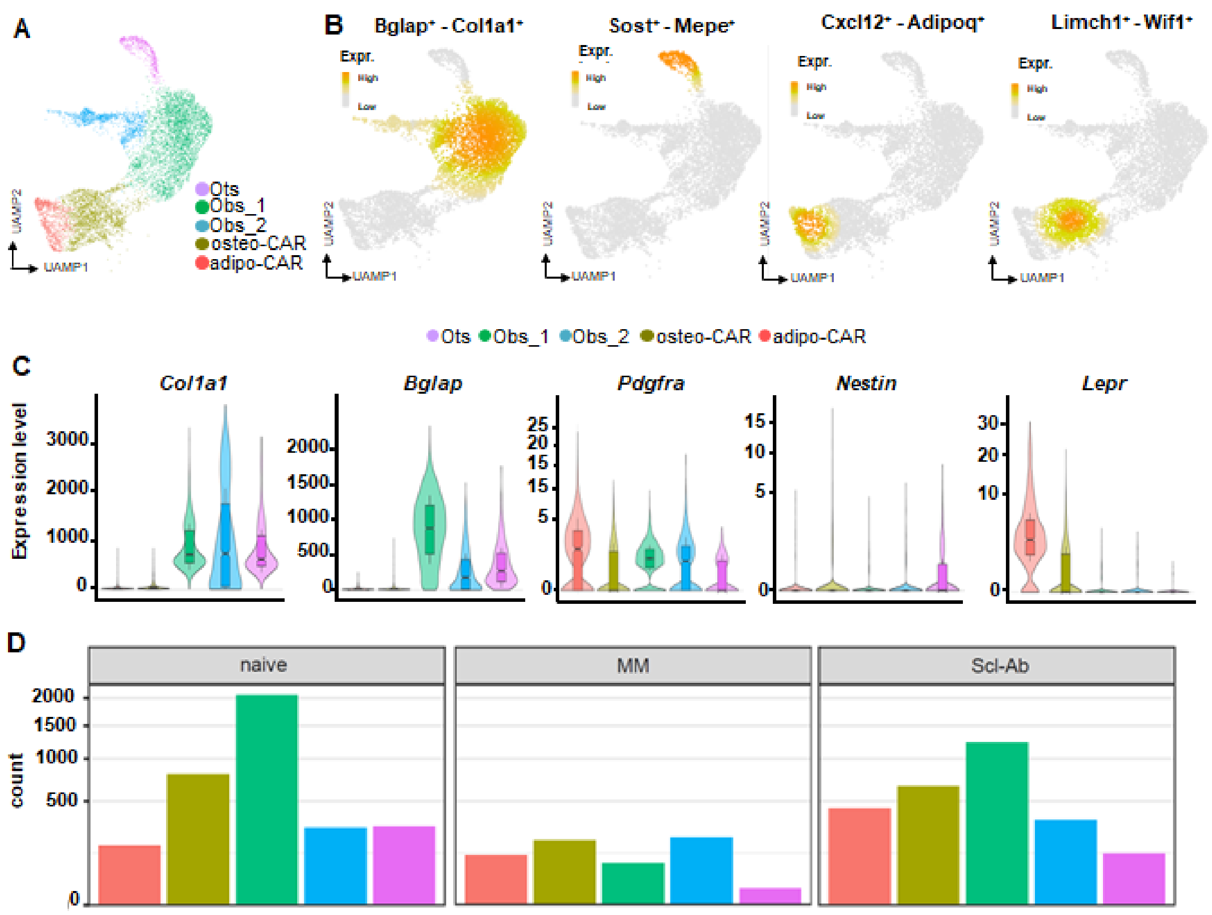

Fig. S7. Identification of osteoblastic cell populations in the scRNAseq dataset. (**A**) Uniform Manifold Approximation and Projection (UMAP) plot representations of the osteoblastic cells detected in the three groups, including the osteocyte, osteoblast-1, osteoblast-2, osteo-CAR, and adipo-CAR cell populations. (**B**) Gene expression demonstrating the identification of each cellular cluster: osteocytes (*Sost*$^{+}$, *Mepe*$^{+}$), osteoblasts_1 and osteoblasts_2 (*Bglap*$^{+}$, *Col1a1*$^{+}$), osteo-CARs (*Limch1*$^{+}$, *Wif1*$^{+}$) and adipo-CARs (*Cxcl12*$^{+,}$ *Adipoq*$^{+}$). The color indicates the intensity of gene expression. (**C**) Relative gene expression (CP10K) of osteoblastic (*Col1a*, *Blgap*) and bone marrow stromal cell (*Pdgfra*, *Nestin*, *Lepr*) markers in each cell cluster. (**D**) Cell counts for each cell cluster for naïve mice, mice bearing MM tumors (MM), and mice treated with Scl-ab.

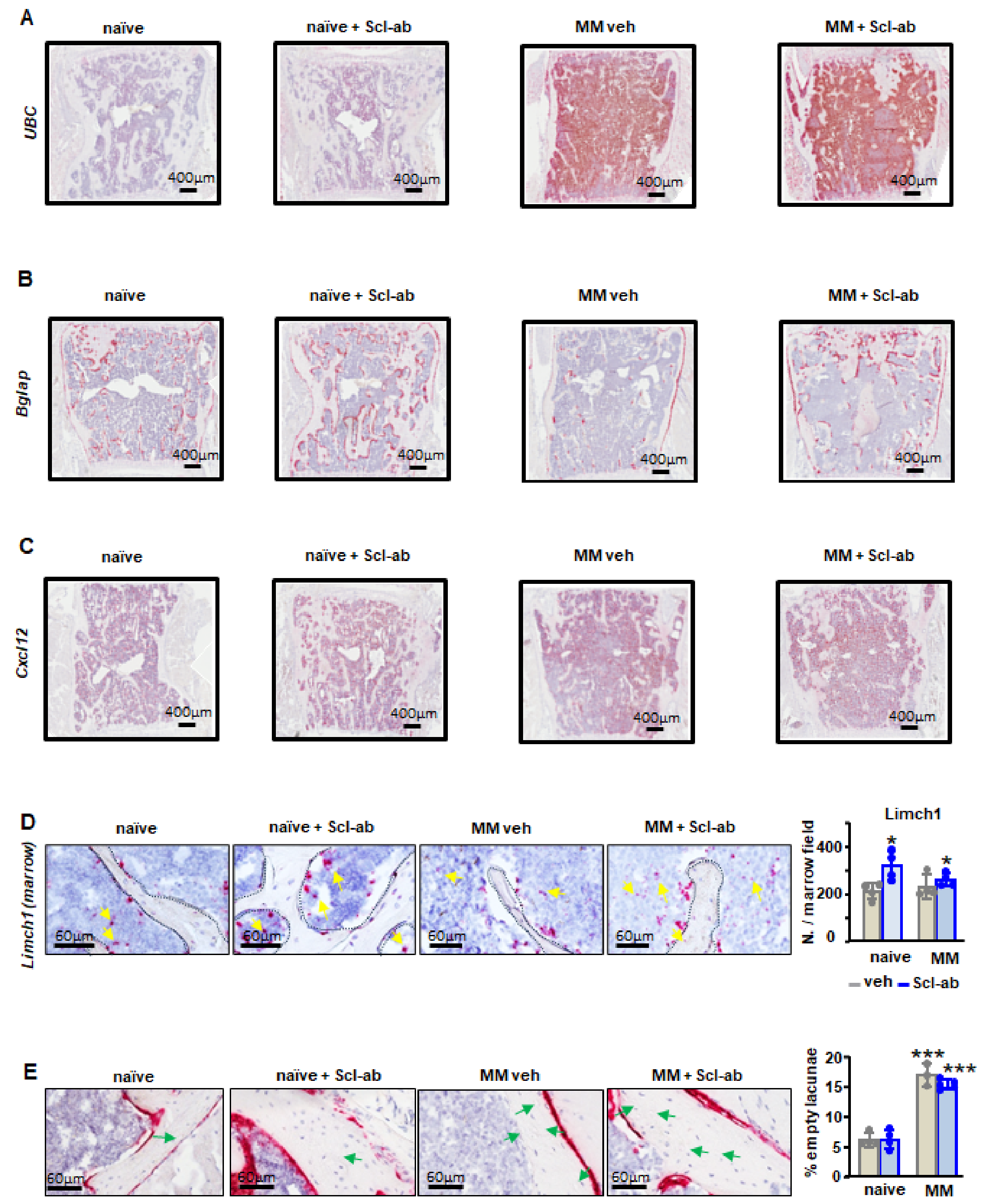

Fig. S8. Histological analysis of osteoblastic populations in bones from naïve mice or MM-bearing mice treated with Scl-ab. RNAscope representative images (**A-C**) of human-UBC and

murine *Bglap*$^+$ and *Cxcl12*$^+$ (scale: 400 µm) in the bone marrow (scale: 60µm). Quantification of (**D**) bone marrow *Limch1*$^+$ cells (scale: 60 µm) and (**E**) empty osteocyte lacunae (scale: 60 µm) in L4-6 vertebrae from control (naïve), naïve mice treated with anti-sclerostin-antibody (Scl-ab), mice human OPM2 MM cells receiving vehicle, or mice bearing murine OPM2 MM cells receiving Scl-ab. n=3-5 bones/group. *p<0.05; **p<0.01; ***p<0.001 vs. naïve vehicle by Two-Way ANOVA (D, E).

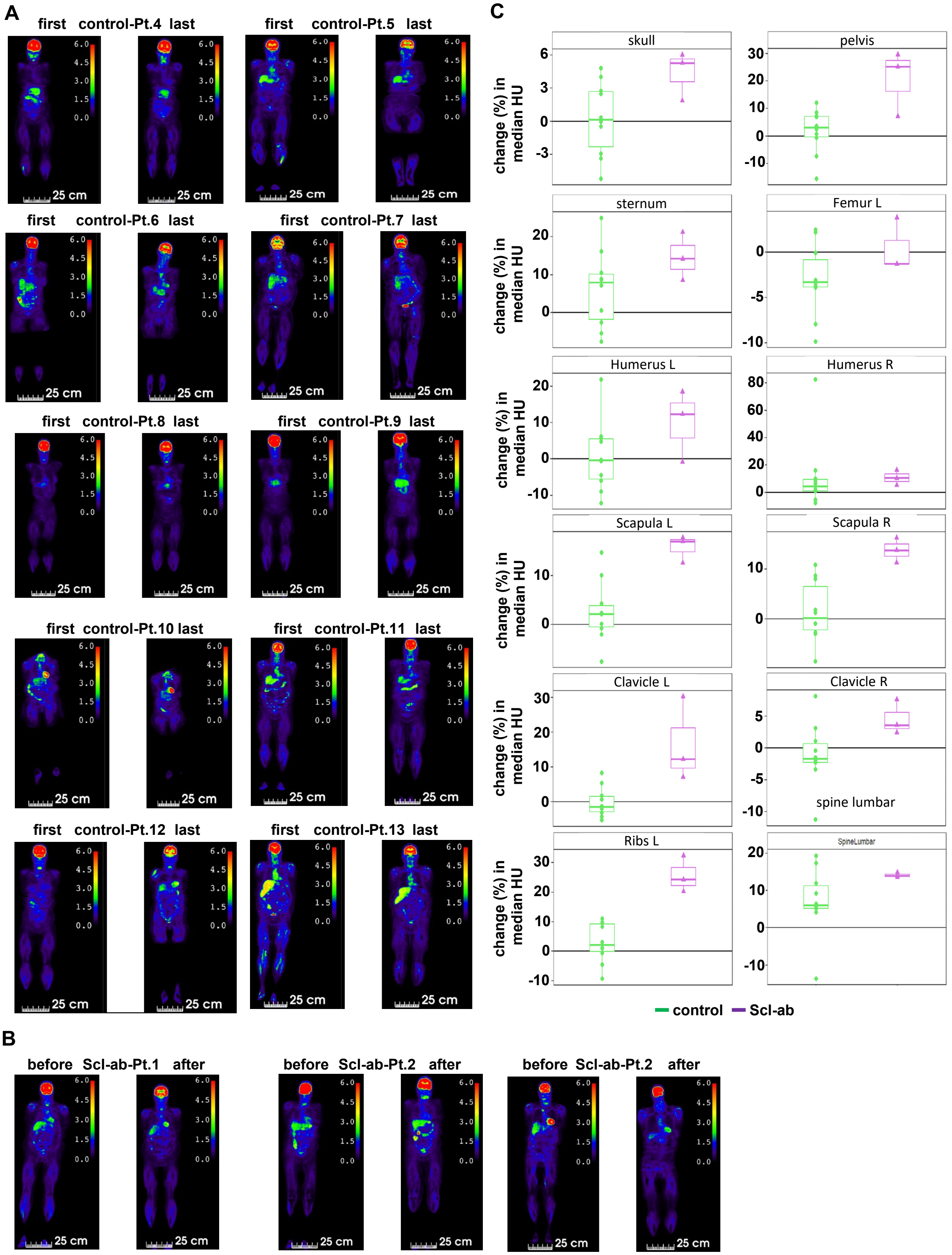
A
first control-Pt.4 last
first control-Pt.5 last
first control-Pt.6 last
first control-Pt.7 last
first control-Pt.8 last
first control-Pt.9 last
first control-Pt.10 last
first control-Pt.11 last
first control-Pt.12 last
first control-Pt.13 last
6.0
4.5
3.0
1.5
0.0
25 cm
B
before Scl-ab-Pt.1 after
before Scl-ab-Pt.2 after
before Scl-ab-Pt.2 after
C
skull
pelvis
sternum
Femur L
Humerus L
Humerus R
Scapula L
Scapula R
Clavicle L
Clavicle R
spine lumbar
Ribs L
SpineLumbar
change (%) in median HU
control
Scl-ab

Fig. S9. Tumor and bone analysis in PET-CT scan from MM patients. (**A**) First and last PET-CT scan of MM patients in remission not receiving Scl-ab. (**B**) PET-CT scans in patients before and after Scl-ab therapy. **(C)** Percent change in median radiodensity Hounsfield Units (HU) in the skull, sternum, humerus, scapula, clavicle, ribs, pelvis, femur, and lumbar spine from patients in remission treated with/without Scl-ab. n=3-10 patients/group.

Parameter	units	saline	MM (veh)	MM (Scl-ab)	MM (BT-GSI)	MM (Bimodal)
ALB	g/dL	4.5 ± 0.3	4.6 ± 0.2	4.4 ± 0.2	4.4 ± 0.2	4.3 ± 0.2
ALP	U/L	77.8 ± 22.3	72.8 ± 22.3	107.0 ± 18.6*	69.8 ± 25.8	80.4 ± 28.1
ALT	U/L	27.5 ± 3.2	23.5 ± 5.0	30.6 ± 7.1	28.0 ± 4.7	40.2 ± 14.4
AMY	U/L	970.1 ± 85.5	958.1 ± 92.9	1083 ± 516.2	926.8 ± 142.1	955.8 ± 155.2
TBIL	mg/dL	0.2 ± 0.04	0.2 ± 0.1	0.2 ± 0.1	0.2 ± 0.0	0.2 ± 0.0
BUN	mg/dL	16.2 ± 3.7	14.4 ± 2.1	16.0 ± 3.2	13.4 ± 3.8	14.0 ± 2.7
CA	mg/dL	10.9 ± 0.2	11 ± 0.6	11.0 ± 0.4	11.2 ± 0.4	10.8 ± 0.3
PHOS	mg/dL	9.1 ± 0.5	9.1 ± 1.4	8.9 ± 1.1	8.8 ± 0.5	9.4 ± 2.1
CRE	mg/dL	0.2 ± 0.04	0.2 ± 0.0	0.2 ± 0.0	0.2 ± 0.0	0.2 ± 0.1
GLU	mg/dL	140.3 ± 28.2	164.4 ± 16.0	172.1 ± 19.3*	163.4 ± 33.3	154.2 ± 5.2
NA+	mmol/L	155.8 ± 3.7	159.4 ± 4.7	159.1 ± 5.7	160.2 ± 4.1	157.4 ± 2.6
K+	mmol/L	8.0 ± 0.5	7.8 ± 0.7	7.6 ± 0.9	8.2 ± 0.4	7.8 ± 1.0
TP	g/dL	6.2 ± 0.3	6.2 ± 0.3	6.4 ± 0.4	5.9 ± 0.3	5.9 ± 0.1*
GLOB	g/dL	1.7 ± 0.3	1.6 ± 0.3	2.1 ± 0.8	1.5 ± 0.2	1.6 ± 0.3
HEM		3 ± 0.0	2.4 ± 0.5*	2.0 ± 1.0*	2.8 ± 0.4	2.2 ± 0.8*

Table S1. Toxicology studies. Serum toxicologic parameters in immunodeficient naïve mice (saline) or mice injected with OPM2 MM cells in the tail vein and treated with vehicle (veh), sclerostin antibody (Scl-ab), bone-targeted Notch inhibitor (BT-GSI)), or Scl-ab + BT-GSI (combo) for three weeks. n=6-11 mice/group. ALB: albumin; ALP: alkaline phosphatase; ALT: alanine aminotransferase; AMY: amylase; TBIL: total bilirubin; BUN: blood urea nitrogen; CA: calcium; PHOS: phosphate; CRE: creatinine; GLU: glucose; NA+: sodium; K+: potassium; TP: total protein; GLOB: globulin; HEM: hemoglobin. *$p<0.05$; **$p<0.01$; ***$p<0.001$ vs. naïve by One-Way ANOVA.

Patient #	Institute	Disease stage	MM therapy	M component (g/dl)		Romo sozumab (Scl-ab)	Anti-resorptive therapy	DXA	PET-CT
				start date	end date				
1	UAMS	remission	Lenalidomide	0	0	Yes	no	Yes	Yes
2	UAMS	remission	no therapy	0	0	Yes	no	Yes	Yes
3	UAMS	remission	VRd	0	0	Yes	no	Yes	Yes
4	UAMS	remission	Ninlaro+Dex	0	0	No	Zometa	Yes	Yes
5	UAMS	remission	no therapy	0	0	No	Zometa	Yes	Yes
6	UAMS	remission	Dara	0	0	No	Zometa	Yes	Yes
7	UAMS	remission	no therapy	0	0	No	Zometa	No	Yes
8	UAMS	remission	Dara+Velcade	0	0	No	no	No	Yes
9	UAMS	remission	no therapy	0	0	No	Zometa	No	Yes
10	UAMS	remission	no therapy	0	0	No	Zometa	No	Yes
11	UAMS	remission	no therapy	0	0	No	Zometa	No	Yes
12	UAMS	remission	no therapy	0	0	No	Zometa	No	Yes
13	UAMS	remission	KRd	0	0	No	Zometa	No	Yes
14	Mayo Clinic	remission	no therapy	≤0.2	0	Yes	no	Yes	No
15	Mayo Clinic	remission	no therapy	0	0	Yes	no	Yes	No

Patient #	BMD T-score DXA (before Scl-ab Rx)	BMD T-score DXA (after Scl-ab Rx)	BMD T-score DXA (change)	DXA Interval (months)	PET-CT Interval (months)
1	-4.4	-3.4	↑ 1.0	38	31
2	-2.4	-2.2	↑ 0.2	25	50
3	-3.9	-2.5	↑ 1.4	30	30
4	-1.8	-2.0	↓0.2	19	24
5	-1.7	-1.6	↑ 0.1	19	31
6	-1.1	-1.4	↓0.3	34	24
7	-	-	-	-	27
8	-	-	-	-	26
9	-	-	-	-	25
10	-	-	-	-	32
11	-	-	-	-	24
12	-	-	-	-	31
13	-	-	-	-	26
14	-2.4	-2	↑ 0.4	30	-
15	-2.4	-1.9	↑ 0.5	12	-

Table S2. Patients' demographics and study characteristics. VRd: Velcade, Revlimid, dexamethasone; Dex: dexamethasone; Dara: daratumumab; KRd: carfilzomib, lenalidomide, dexamethasone. M: male; F: female. The M component was determined at the start and the end of the treatment. DXA measurements were taken before (initial) and after Scl-ab treatment.

References

1. Sabol HM, Ashby C, Adhikari M, Anloague A, Kaur J, Khan S, et al. A NOTCH3-CXCL12-driven myeloma-tumor niche signaling axis promotes chemoresistance in multiple myeloma. Haematologica. 2024.
2. Sabol HM, Ferrari AJ, Adhikari M, Amorim T, McAndrews K, Anderson J, et al. Targeting Notch Inhibitors to the Myeloma Bone Marrow Niche Decreases Tumor Growth and Bone Destruction without Gut Toxicity. Cancer Res. 2021;81(19):5102-14.
3. Kaur J, Adhikari M, Sabol HM, Anloague A, Khan S, Kurihara N, et al. Single-Cell Transcriptomic Analysis Identifies Senescent Osteocytes That Trigger Bone Destruction in Breast Cancer Metastasis. Cancer Res. 2024;84(23):3936-52.
4. Delgado-Calle J, Anderson J, Cregor MD, Condon KW, Kuhstoss SA, Plotkin LI, et al. Genetic deletion of Sost or pharmacological inhibition of sclerostin prevent multiple myeloma-induced bone disease without affecting tumor growth. Leukemia. 2017;31(12):2686-94.
5. Nookaew I, Papini M, Pornputtapong N, Scalcinati G, Fagerberg L, Uhlén M, et al. A comprehensive comparison of RNA-Seq-based transcriptome analysis from reads to differential gene expression and cross-comparison with microarrays: a case study in Saccharomyces cerevisiae. Nucleic Acids Res. 2012;40(20):10084-97.
6. Dobin A, Davis CA, Schlesinger F, Drenkow J, Zaleski C, Jha S, et al. STAR: ultrafast universal RNA-seq aligner. Bioinformatics. 2013;29(1):15-21.
7. Quinlan AR, Hall IM. BEDTools: a flexible suite of utilities for comparing genomic features. Bioinformatics. 2010;26(6):841-2.

8. Liu R, Holik AZ, Su S, Jansz N, Chen K, Leong HS, et al. Why weight? Modelling sample and observational level variability improves power in RNA-seq analyses. Nucleic Acids Res. 2015;43(15):e97.

9. Ritchie ME, Phipson B, Wu D, Hu Y, Law CW, Shi W, et al. limma powers differential expression analyses for RNA-sequencing and microarray studies. Nucleic Acids Res. 2015;43(7):e47.

10. Stuart T, Butler A, Hoffman P, Hafemeister C, Papalexi E, Mauck WM, 3rd, et al. Comprehensive Integration of Single-Cell Data. Cell. 2019;177(7):1888-902.e21.

11. Hao Y, Hao S, Andersen-Nissen E, Mauck WM, 3rd, Zheng S, Butler A, et al. Integrated analysis of multimodal single-cell data. Cell. 2021;184(13):3573-87.e29.

12. Hafemeister C, Satija R. Normalization and variance stabilization of single-cell RNA-seq data using regularized negative binomial regression. Genome Biol. 2019;20(1):296.

13. Lancichinetti A, Fortunato S. Community detection algorithms: A comparative analysis. Physical Review E. 2009;80(5):056117.

14. Nookaew I, Xiong J, Onal M, Bustamante-Gomez C, Wanchai V, Fu Q, et al. Refining the identity of mesenchymal cell types associated with murine periosteal and endosteal bone. J Biol Chem. 2024;300(4):107158.

15. Väremo L, Nielsen J, Nookaew I. Enriching the gene set analysis of genome-wide data by incorporating directionality of gene expression and combining statistical hypotheses and methods. Nucleic Acids Res. 2013;41(8):4378-91.

16. Mann DCR, M.; Farmer, P.; Eichhorn, J.; Manzil, F.F.P., Wardell, C.P. Evaluating Skellytour for Automated Skeleton Segmentation from Whole-Body CT Images. Radiology: Artificial Intelligence. 2025;in press.

17. Kenward MG, Roger JH. Small sample inference for fixed effects from restricted maximum likelihood. Biometrics. 1997;53(3):983-97.

18. Holm S. A Simple Sequentially Rejective Multiple Test Procedure. Scandinavian Journal of Statistics. 1979;6(2):65-70.
